# Differential regulation of triterpene biosynthesis induced by an early failure in cuticle formation in apple

**DOI:** 10.1038/s41438-021-00511-4

**Published:** 2021-04-01

**Authors:** Luigi Falginella, Christelle M. Andre, Sylvain Legay, Kui Lin-Wang, Andrew P. Dare, Cecilia Deng, Ria Rebstock, Blue J. Plunkett, Lindy Guo, Guido Cipriani, Richard V. Espley

**Affiliations:** 1 Dipartimento di Scienze Agroalimentari, Ambientali e Animali, University of Udine, Udine, Italy; 2 Research Center, Vivai Cooperativi Rauscedo, Rauscedo, Italy; 3 The New Zealand Institute for Plant and Food Research, Auckland, New Zealand; 4 The Luxembourg Institute of Science and Technology, Esch-sur-Alzette, Luxembourg

**Keywords:** Abiotic, Secondary metabolism

## Abstract

Waxy apple cuticles predominantly accumulate ursane-type triterpenes, but the profile shifts with the induction of skin russeting towards lupane-type triterpenes. We previously characterised several key enzymes in the ursane-type and lupane-type triterpene pathways, but this switch in triterpene metabolism associated with loss of cuticle integrity is not fully understood. To analyse the relationship between triterpene biosynthesis and russeting, we used microscopy, RNA-sequencing and metabolite profiling during apple fruit development. We compared the skin of three genetically-close clones of ‘Golden Delicious’ (with waxy, partially russeted and fully russeted skin). We identified a unique molecular profile for the russet clone, including low transcript abundance of multiple cuticle-specific metabolic pathways in the early stages of fruit development. Using correlation analyses between gene transcription and metabolite concentration we found MYB transcription factors strongly associated with lupane-type triterpene biosynthesis. We showed how their transcription changed with the onset of cuticle cracking followed by russeting and that one factor, MYB66, was able to bind the promoter of the oxidosqualene cyclase OSC5, to drive the production of lupeol derivatives. These results provide insights into the breakdown of cuticle integrity leading to russet and how this drives MYB-regulated changes to triterpene biosynthesis.

## Introduction

Many epidemiological studies have found a reduced risk of various chronic diseases associated with the consumption of apple^1^. The most influential classes of apple-derived bioactives are the polyphenols (including phenolic acids, anthocyanins, flavan-3-ols, dihydrochalcones and flavonols), pentacyclic triterpenes and ascorbic acid^2–4^. Pentacyclic triterpenes possess numerous biomedical properties^5^, including anti-inflammatory^2^, anti-cancer^6^ and anti-plasmodial activities^7^. In apple skin, triterpenes accumulate to a similar extent to phenolic compounds, with concentrations ranging from 825 to 6707 μg g^−1^ fresh weight (FW) for triterpenes and 733 to 4868 μg g^−1^ FW for phenolics. Ursolic and oleanolic acids, of the ursane and oleanane triterpene types, predominate in the skins of most (waxy) commercial apple varieties^2^. Conversely, suberized apple skin tissue, found in partially and fully russeted heritage apple varieties, contains higher concentrations of lupane derivatives, including betulinic acid and specific triterpene esters such as betulinic acid-3-trans-caffeate, a pharmaceutically potent triterpene-hydroxycinnamate^8^.

In apple fruit, triterpenes occur in the cuticular layer of the skin, where they can constitute up to 60% of the total wax content^9^. The cuticle is composed of cutin polymers, which are embedded and overlaid by soluble cuticular waxes^10^. Cuticular wax is a mixture of very-long-chain fatty acids, their esters and derivatives, including alkanes, aldehydes, primary and secondary alcohols, ketones, and specialised metabolites, such as triterpenes and phenolic compounds. The physiological function of the cuticle is to protect the epidermal layer and the underlying internal fruit flesh from biotic and abiotic stresses^11^. Fruit cuticle biosynthesis has been shown to be positively regulated by hormonal (abscisic acid (ABA)), developmental and environmental factors^12,13^.

When the cuticle is severely compromised, russeting can occur^14^. Russeting involves the accumulation of suberin in the inner part of the cell wall of the epidermal cell layer. The formation of microcracks in the cuticle is generally considered as a key trigger for apple russeting^15^. Fruit exposure to extreme growth conditions at the early stages of development and genetic factors are responsible for microcrack development. These fissures increase the cuticle’s water permeability and trigger the formation of ‘repair’ patches of secondary skin (periderm) that replace the primary skin (epidermis and hypodermis)^16,17^. While the presence of russet may have beneficial flavour notes^18^ and unusual health-promoting properties^8^, this disorder compromises fruit appearance and thus, its commercial value. It is also associated with reduced postharvest properties, a major one being higher water loss^17,19^.

Suberin differs from cutin in having longer chain fatty acids with a phenolic domain and tends to have less elasticity and more water permeability^20^. A bulk transcriptomic analysis of three waxy and three russeted apple varieties showed that the expression of cuticle biosynthetic genes (cutin and wax) in fully russeted varieties is significantly lower than those of their non-russeted counterparts^21^. To identify genetic factors controlling cuticle in apple, two quantitative trait locus (QTL) mapping surveys have been conducted. Falginella et al.^14^ identified a major genetic determinant for russeting on chromosome 12 with an ABC transporter (ABCG11) as a candidate for cutin formation. Lashbrooke et al.^22^ highlighted an ethylene response subfamily member, MdSHN3, on chromosome 15 as a key regulator of cuticle synthesis. A MYB transcription factor (TF), MdMYB93, has also been identified as a master regulator controlling genes involved in suberin biosynthesis, transport and deposition^23^. This study also uncovered a number of triterpene-associated genes and it was postulated that MdMYB93 may be associated with triterpenoid regulation. A recent QTL analysis performed on a cross between ‘Royal Gala’ and ‘Granny Smith’ reported that the genetic control of triterpenes in apple skin is polygenic and that the apple chromosomes with the most QTLs were linkage group (LG)3, LG5, LG9 and LG17^24^.

Pentacyclic triterpenes are formed by the cyclization of 2,3-oxidosqualene to produce ursane-type, oleanane-type and lupane-type triterpenes: ursolic acid, oleanolic acid and betulinic acid from α-amyrin, β-amyrin and lupeol, respectively. This cyclization is performed by oxidosqualene cyclase (OSC) genes. These include OSC1 and OSC3, which are mixed amyrin synthases producing α-amyrin and β-amyrin (in a ratio of 5:1 in transient assays); OSC4, which primarily produces an oleanane triterpene, as well as β-amyrin and lupeol; and OSC5, which produces lupeol primarily and β-amyrin^25,26^. A P450, CYP716A175, was shown to produce ursolic acid, oleanolic acid and betulinic acid from the C-28 oxidation of α-amyrin, β-amyrin and lupeol, respectively^25^. Interestingly, gene expression of OSC1 was shown to correlate with ursolic/oleanolic acid, while OSC5 expression correlated with betulinic acid. This specific transcription profile suggests that these OSC genes may be regulated by TFs to produce the different end products according to environmental or development stimuli.

In this study, we used ‘Golden Delicious’ (GD) apple, with little to moderate skin russet, and compared it with two of its mutational sports, known as ‘Smoothee’ (SM) and ‘Rugiada’(RU)^27^. As sports, these varieties are near identical at the genetic level. However, RU is heavily and consistently russeted over most of the fruit surface, while SM rarely shows any russet. As such, they offer suitable models to test the genetic basis of russeting. In this study, we focused on metabolic pathway switches between russet and non-russet fruit skin. Based on targeted metabolite analysis and qPCR analysis of key cutin/wax and suberin gene transcriptional activity from a detailed sampling regime, we chose three-time points for RNA-seq analysis. Contrasting expression of drought-responsive and ABA-responsive genes illustrated the important role of ABA signalling on russet development. Canonical correlation analysis on metabolite and transcript data highlighted key MYB TFs involved in the triterpene shift observed in russeted apple skin. Of these, MYB52 and MYB66 were able to activate the promoter of OSC5, suggesting a regulatory role in the production of lupane-type triterpenes.

## Results

### Russet and cuticle defects occur at the exponential growth phase

**Fig. 1:**
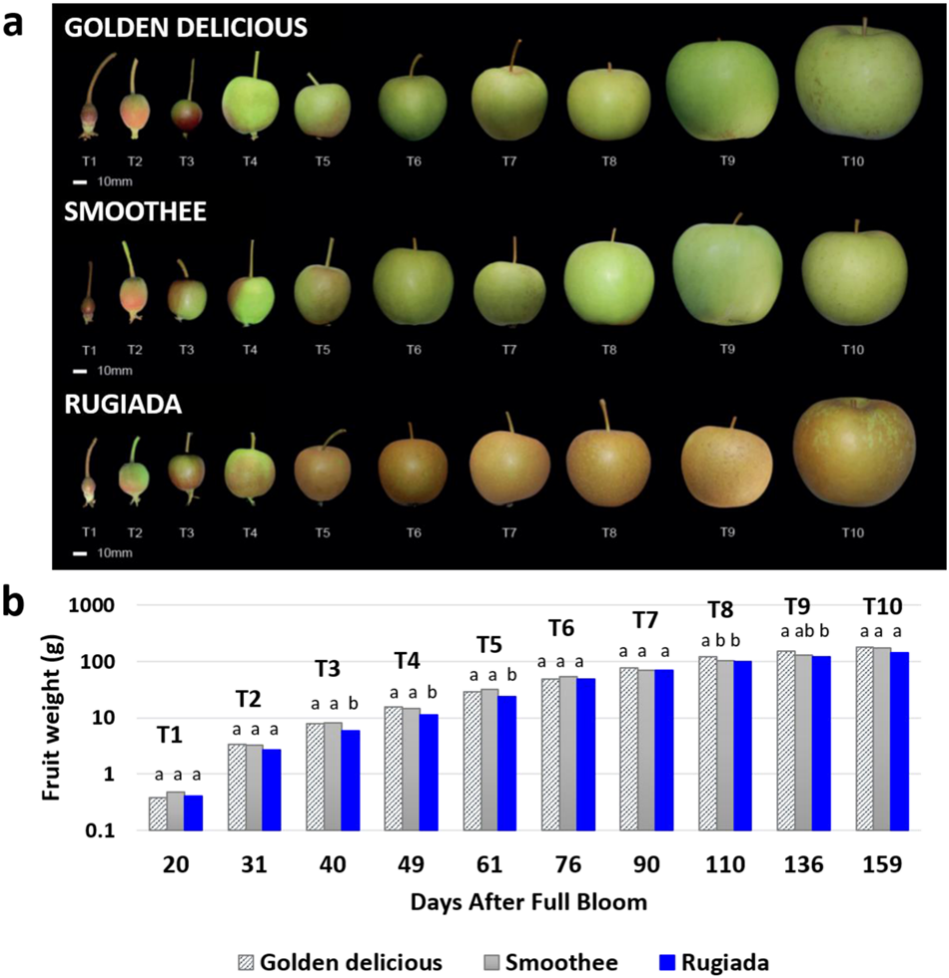
Fruit growth of the three phenotypically different apple varieties. **a** ‘Golden Delicious’, and its fully russeted and russet-resistant mutational sports, ‘Rugiada’ and ‘Smoothee’. **b** Evolution (logarithmic scale) of the fruit weight during growth. Significance was calculated according to one-way ANOVA of *p* < 0.05 per time point, where lowercase letters above bars result from the comparison of groups using Tukey’s test

**Fig. 2:**
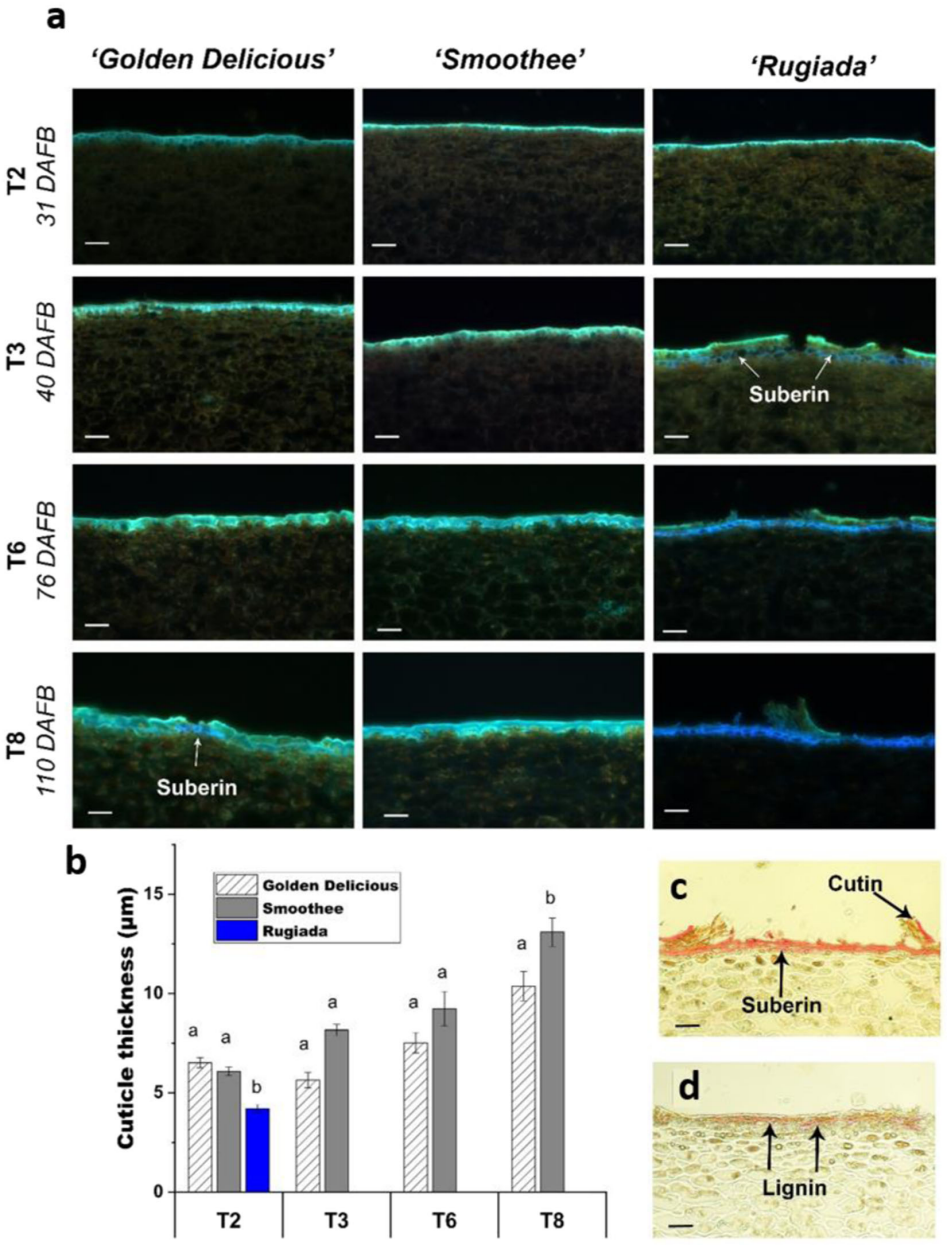
Light microscopy analysis of the epidermal cell layer of the three apple genotypes. **a** Cross-sections of the epidermal layer of ‘Golden Delicious’ and its russet and non-russet clones, ‘Rugiada’ and ‘Smoothee’, showing autofluorescent structures. Flavonoids are mainly responsible for the fluorescence of the cuticle (green). The presence of suberin and/or lignin (blue emission) appears from T3 in ‘Rugiada’ and T8 in ‘Golden Delicious’. Excitation 355 nm with emission at 400–800 nm. Scale bar = 50 µm. **b** Cuticle thickness measured using the lipid stain Sudan IV. The cuticle of the russet clone ‘Rugiada’, although intact at T2, shows a significantly reduced thickness as compared to ‘Golden Delicious’ and ‘Smoothee’. Significance was calculated according to a one-way ANOVA of *p* < 0.05. The cuticle of ‘Rugiada’ could not be measured after T2. **c** Light microscopy of the epidermal layer of T6 stage fruit from ‘Rugiada’ using the lipid stain Sudan IV shows the presence of suberin in the periderm and the dramatic reduction in cuticle deposition (with only patches of cutin remaining). **d** Phloroglucinol staining of the T8 stage fruit from ‘Rugiada’ showing lignified cell-wall tissue in the periderm (pink and red)

Fruit from GD apple and its russet-resistant mutational sport SM showed similar growth rates during the 10 fruit developmental stages. For the fully russeted sport, RU, we observed a slight delay in fruit growth between 40 and 76 DAFB (Fig. 1a, b). The highest increase in fruit weight occurred between T1 (20 DAFB) and T3 (40 DAFB), likely corresponding to a period of high mechanical strain for the skin. It was also at 40 DAFB that browning/russeting of RU fruit skins occurred, whereas no trace of russeting was visible prior to this. Autofluorescence microscopy analysis of fruit skins confirmed that the integrity of RU cuticle was maintained at 31 DAFB (Fig. 2a), although its cuticle layer (made of cutin and wax) was thinner than the ones of its counterparts (Fig. 2b). At 40 DAFB, the RU cuticle displayed microcracking between epidermal cells, with the formation of a secondary fruit surface (periderm), characterised by suberin and lignin deposition (Fig. 2a, b, c and Fig S1). A few patches of russeting appeared in the calyx area of GD fruit from 76 DAFB, whereas SM fruit skin remained intact throughout development (Figs. 1a, 2a).

### Triterpene and phenolic profiles change with the onset of russeting

**Fig. 3:**
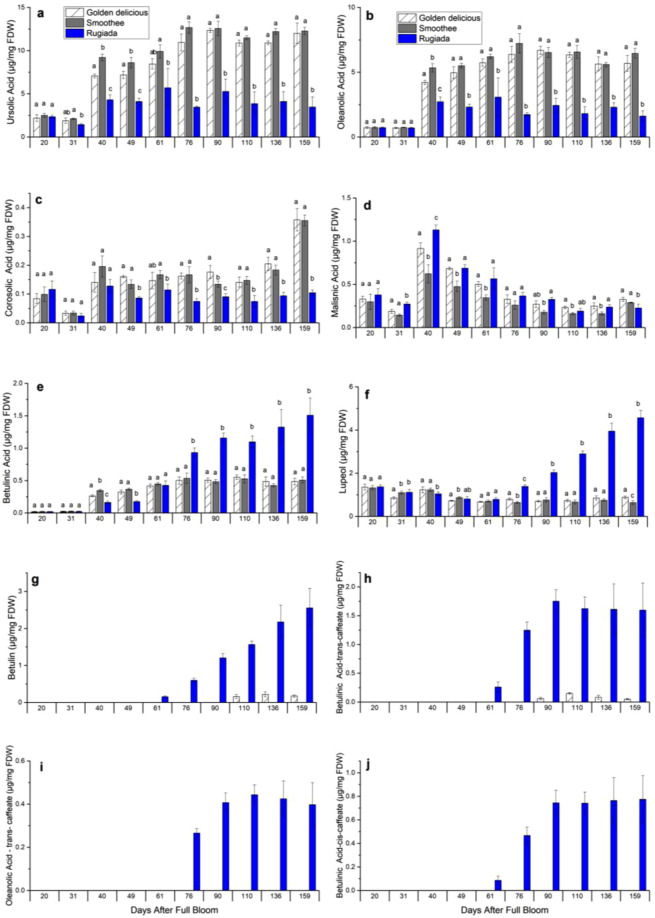
Triterpene contents in ‘Golden Delicious’, ‘Rugiada’ and ‘Smoothee’. Data represent means and SD of three biological replicates each per sample (generated from a pool of skin tissues from six to nine different fruits). Significance was calculated according to one-way ANOVA of *P* < 0.05 per time point, where lowercase letters above bars result from the comparison of groups using Tukey’s test (for triterpenes **a** to **j**). Groups connected by the same letter are not significantly different. Data are expressed in µg per mg of freeze-dried weight. Values at 20 and 31 DAFB are expressed per mg of total fruit and not skin, as the fruits were too small at this stage to peel them consistently

Pentacyclic triterpenes of the ursane-series and oleanane-series are the predominant cutin-associated wax component in primary ‘waxy’ apple cuticles^25,28,29^. In this study, the skin concentrations in ursolic and oleanolic acids were significantly lower in RU as compared to GD and SM (*p* < 0.05) from 40 DAFB and thereafter, corroborating the decreased cuticle layer observed for the russet mutant in microscopy at 31 DAFB and its scattered presence at the following time points (Fig. 3a,b). Their precursors, α-amyrin and β-amyrin, were not detected. The hydroxylated version of ursolic acid, corosolic acid, followed the same trend during fruit growth (Fig. 3c), whereas that of oleanolic acid, maslinic acid (Fig. 3d) decreased through the development and was similar in all three genotypes. In previous studies on russet apple varieties, the presence of a suberized secondary skin was linked to betulinic acid accumulation in the wax^28^. Similarly in our study, RU tissues presented higher levels of most lupane-type triterpenes (*p* < 0.05) from 76 DAFB, including betulinic acid (Fig. 3e), lupeol (Fig. 3f), betulin (Fig. 3g), as well as of triterpene-caffeate derivatives (Fig. 3h, i, j) as compared to GD and SM skin tissues.

Phenolic profiling of the apple skin extracts revealed the presence of five main families of compounds: flavan-3-ols, procyanidins, flavonols, dihydrochalcones, and hydroxycinnamic acids (Fig. S2 and Table S1). At the key time point showing the transition from primary to secondary skin development (40 DAFB), RU skin significantly accumulated fewer flavonols (quercetin derivatives) and more dihydrochalcones (including phloridzin) and phenolic acids compared with GD and SM. In all three varieties tested, the most striking observation was the sharp reduction in the majority of phenolic compounds between 40 and 49 DAFB, which is in agreement with previous literature^30–32^. During later developmental stages, the most noticeable difference was in the relative increase of procyanidins (when considered as a total) in RU compared to either GD or SM, whereas no difference was observed in the flavan-3-ol monomer content (catechin and epicatechin), suggesting an increased polymerisation rate or procyanidin storage in RU skin.

### Expression of OSC genes and triterpene patterning

**Fig. 4:**
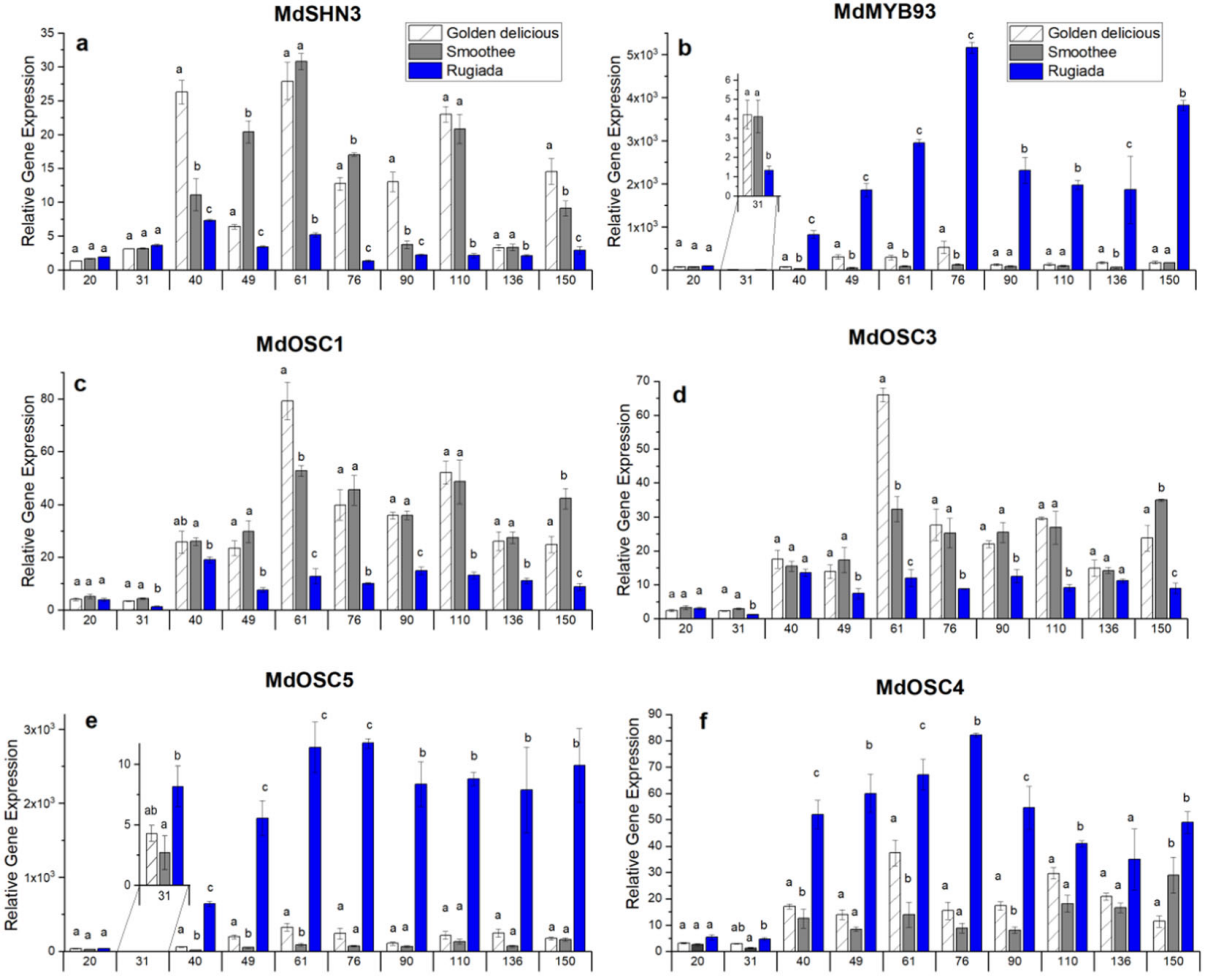
Differential expression analysis. **a** Cutin/wax regulation (MdSHN3), **b** suberin regulation (MdMYB93) and **c**–**f** triterpene biosynthesis (MdOSC1-5). Analysis was performed on RNA extracted from apple skin tissues. Data represent means and SE of three biological replicates (generated from a pool of skin tissues from four different fruits). Significance was calculated according to one-way ANOVA for each time point (*p* < 0.05), where lowercase letters above bars result from the comparison of groups using Tukey’s. Groups connected by the same letter are not significantly different

The expression of key cuticle-related genes was analysed over the developmental series. The transcription abundance of MdSHN3, a regulator of fruit cuticle assembly in apple^22^ was significantly lower in RU from 40 DAFB as compared to GD and SM (Fig. 4a), in agreement with the loss of integrity of its cuticle (Fig. 2). The expression of MdMYB93, a TF involved in suberin deposition^23^, showed increased expression in RU from 40 DAFB (Fig. 4b), concomitant with suberin occurrence (Fig. 2a). The two OSC genes (MdOSC1 and MdOSC3) known to produce the cutin-related ursane and oleanane-type of triterpenes, α-amyrin and β-amyrin, were downregulated in RU from 31 DAFB (Fig. 4c, d), while MdOSC5, which has been shown to preferentially convert 2,3-oxidosqualene into lupeol prior to conversion into betulinic acid, was highly upregulated from 31 DAFB in RU as compared with GD and SM (Fig. 4e). MdOSC1 and MdOSC3 followed the same trend for the accumulation of ursane and oleanane-type triterpenes (Fig. 3), while a delay was observed between the increase in lupane-type triterpene accumulation (76 DAFB) and the upregulation of MdOSC5 (31 DAFB) in RU. This discrepancy could be explained by the fact that lupeol is also produced as a minor component through MdOSC1/3/4 enzymatic activities^25^. MdOSC4, which predominantly converts 2,3-oxidosqualene into germanicol (before conversion to morolic acid), showed higher expression between 40 and 110 DAFB in RU (Fig. 4f). Since no germanicol derivatives were detected in the free wax fractions of skin tissues, the role played by MdOSC4 remains unclear.

### Genes associated with cuticle formation and russeting

Using data from gene expression and the shift in triterpene profiles (Figs. 3 and 4), we chose to perform RNA sequencing for GD and RU at three-time points (40, 76, and 110 DAFB). Although GD skin presented slight russeting in later developmental stages, it showed similar expression and metabolite abundance data to SM. A total of 18 cDNA libraries were sequenced and mapped on the reference genome GDDH13^33^. A mapping rate of 97.5% to 99.5% was obtained with 45,116 genes identified. Principal component analysis (PCA) of the transcriptome data (RPKM) showed major transcriptional changes between russeted and non-russeted tissues, as well as an impact of the developmental stage, with a clear separation between gene expression at 40 DAFB and at 76 and 110 DAFB (Fig. S3). Using stringent cut-off statistical values (p adj. <0.01 and −1 > log2 (FC) > 1), 4495 differentially expressed genes (DEG) were detected. The number of significantly upregulated genes was 2.3× higher than the number of downregulated genes (3148 and 1347 genes, respectively) (Fig. S3). This unusual trend has previously been described in russeted apple tissue^21,34^, where the suberin pathway has been activated. A total of 758 DEGS were consistently differentially expressed among the three stages and were used to highlight the most affected pathways.

On the basis of candidate genes presented in previous reports^16,34,35^ and KEGG pathways, a schematic heatmap reconstituting cutin, wax, suberin, phenylpropanoid and tri(terpenoid) pathways and their associated gene expression was built, providing a unique tool to identify cuticle-related candidate genes in apple (Fig. S4). Several cutin-related and wax-related genes were significantly downregulated from 40 DAFB, including genes from the fatty acid elongation process, cytochrome P450 86A4 (CYP86A4), long-chain acyl-CoA synthetases (LACSs), fatty acid hydroxylases (CER1). 3-Hydroxy-3-methylglutaryl-coenzyme A reductase (HMGR) involved early in the triterpene (wax) pathway (cytosolic mevalonate pathway) was also drastically reduced in RU. The picture was able to be further completed using recently published genome-wide analyses of cuticle-related genes in apple (Table S2). Our contrasting data set allowed the identification of members of these families that are directly related to cuticle formation and integrity, including LACS gene models^36^, β-Ketoacyl-CoA synthases (KCSs)^37^, GDSL-type esterases/lipases (GELPs)^38^, ABC-G transporters^39^ or in suberin assembly (BAHD acyltransferases^40^). As an example, KCS is the key rate-limiting enzyme in the elongation process of very-long-chain fatty acids (VLCFAs) constituting plant wax^37^. MdKCS2, MdKCS6, MdKCS11, MdKCS15 (from the FDH-like subgroup) and MdKCS21-MdKCS26 (CER6) were all downregulated in RU, emphasizing their involvement in apple wax synthesis. On the other hand, MdKCS19 and MdKCS8 were upregulated in RU and could be associated with suberin-related wax formation.

### Candidate MYB regulators associated with triterpene biosynthesis

**Fig. 5:**
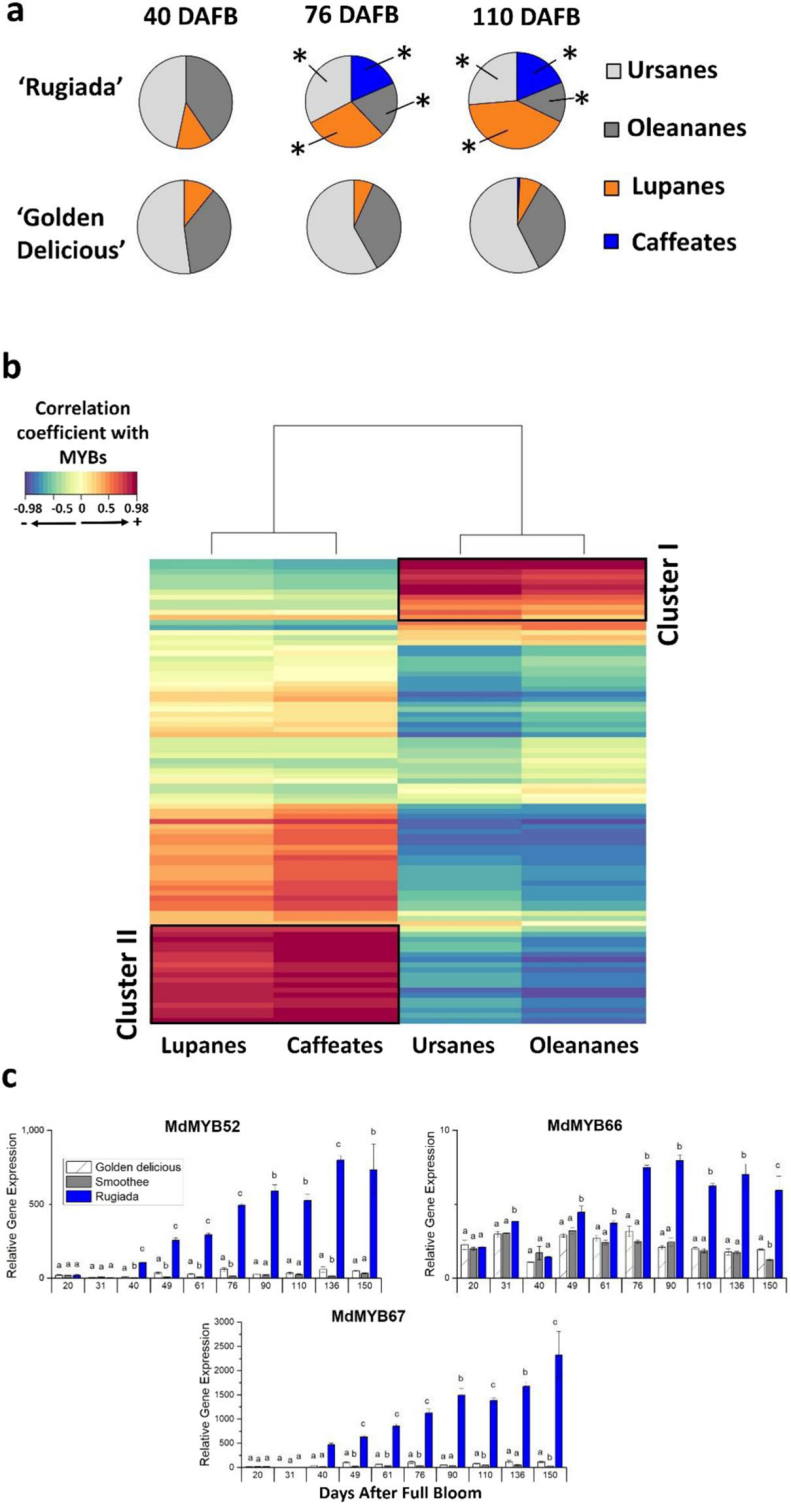
Profiles in triterpenes ‘Rugiada’ and ‘Golden Delicious’ at the three developmental stages selected for RNA-sequencing analysis. **a** A shift in triterpene pattern in ‘Rugiada’ is noticeable from 76 Days after Full Bloom (DAFB), where lupane-type triterpenes and caffeate conjugate proportion increase. *Significant differences (*p* ≤ 0.01) between ‘Rugiada’ and ‘Golden Delicious’ concentrations, as determined by Student *t*-tests. **b** Heat map representing the correlation coefficients between triterpene concentrations (classified per type) and the expression of genes in the MYB family (RPKM values). The heat map was calculated using normalised log10-transformed metabolite/gene expression levels, mean-centered, and standardised data. Cluster I represents a subset of MYB genes that were highly correlated with both the ursane-type and oleanane-type triterpenes, while cluster II represents a subset of MYB genes that were highly related to the lupane series of triterpenes, as well as to the triterpene-caffeates. See Table 1 for gene descriptions. **c** Differential expression analysis of genes identified in cluster II over the developmental series. Data represent means and SE of three biological replicates each per sample (generated from a pool of skin tissues from four different fruits). Significance was calculated according to one-way ANOVA for each time point (*p* < 0.05), where lowercase letters above bars result from the comparison of groups using Tukey’s. Groups connected by the same letter are not significantly different

From RNA-seq data we identified several TFs that were potentially involved in the shift towards the lupane-type triterpene metabolic pathway (Fig. 5a). TFs from the R2-R3 MYB, WRKY, basic helix–loop–helix (bHLH) and ethylene-responsive factor (ERF) classes were differentially expressed in RU compared with GD. Of particular interest were MYB TFs as some MYBs have been linked to the regulation of both cuticle^41^ and terpene biosynthesis^42,43^ and, in apple, triterpenes are an integral part of cutin-associated and suberin-associated waxes^28^. A regularised canonical correlation analysis (rCCA) was performed to identify MYBs that were related to the production of specific types of triterpenes. In total, 91 differentially expressed MYBs were included in the analysis (Table S3). Two distinct gene clusters were highlighted (Fig. 5b): one containing MYBs highly related to ursane-type and oleanane-type triterpenes, and the second composed of MYBs correlating with lupane-type and triterpene-caffeates.

Five gene models orthologous to MYB16, MYB17, MYB94 were strongly linked with cutin-associated triterpenes (ursanes and oleananes) (Table 1), indicating their potential involvement in cuticular wax synthesis. Among the 14 candidate MYB genes associated with lupanes and triterpene-caffeates, MYB52 (MD05G1011100 and MD10G1010900), MYB66 (MD09G1183800), and MYB67 (MD05G1239200) have been associated with suberin biosynthesis in the literature. Two gene models, orthologous to MYB52 (MD05G1011100 and MD10G1010900), were strongly correlated with the suberin-associated triterpenes.

**Table 1:** MYB genes observed in cluster I from Fig. 5

				Log2 fold change (RU/GD)		
Gene ID	Gene symbol	Annotation description	Sequence Length	T3	T6	T8
*a. Cluster I*						
MD09G1054000	MYB16 (MdMYB29)	myb domain protein 16 (AtMYB16, AtMIXTA)	1755	−1.33	−2.59	−2.15
MD17G1050900	MYB94 (MdMYB30-like)	myb domain protein 94 (AtMYB94)	1553		−1.91	−1.84
MD01G1054800	MYB17 (MdMYB14)	myb domain protein 17 (AtMYB17)	1889		−2.58	−1.92
MD17G1051700	MYB16 (MdMYB29-like)	myb domain protein 16 (AtMYB16, AtMIXTA)	1829	−1.42	−1.99	−1.52
MD06G1192900	MYB94 (MdMYB31)	myb domain protein 94 (AtMYB94)	1754	−1.31	−2.90	−1.26
*b. Cluster II (r* *>* *0.89)*						
MD15G1302300	MYB84/68	myb domain protein 84/68 (AtMYB84/68)	1140		1.68	1.10
MD02G1191000	MYB68/84	myb domain protein 68/84 (AtMYB68/84)	1160		2.17	1.55
MD04G1092400	MYB36	myb domain protein 36 (AtMYB36)	1515			1.13
MD09G1183800	MYB66	myb domain protein 66 (AtMYB66, WEREWOLF, WER1, MYB66)	1194		1.16	1.36
MD13G1026400	MYB6	myb domain protein 6 (AtMYB6, MYB6)	1096	2.41	2.40	3.64
MD10G1124100	MYB4 (MdMYB16)	myb domain protein 4 (AtMYB4)	1413	0.65	1.35	1.61
MD10G1304600	MYB102 (MdMYB36-like)	MYB-like 102 (AtMYB102)	1340	1.66	2.29	2.50
MD05G1239200	MYB67 (MdMYB43-like)	myb domain protein 67 (AtMYB67)	1068	3.41	2.29	3.10
MD10G1010900	MYB52	myb domain protein 52 (AtMYB52)	1503	2.43	2.72	3.18
MD05G1011100	MYB52-like	myb domain protein 52 (AtMYB52)	1468	1.64	2.72	2.77
MD16G1010100	Unknown MYB	myb-like transcription factor family protein	1352	2.32	3.38	3.11
MD13G1013200	Unknown MYB	myb-like transcription factor family protein	1735		1.22	1.18
MD02G1076300	Unknown MYB	myb-like transcription factor family protein	1196		1.96	3.37
MD15G1204700	Unknown MYB	myb-like transcription factor family protein	1326		1.74	1.44

Genes are directly related to the concentration of lupane-type triterpenes, i.e., the sum of all lupeol derivatives) a and in cluster II (genes correlated (*r* > 0.85) to the concentrations of ursane-series and oleanane-series of triterpenes (sum of all α-amyrin and β-amyrin derivatives, respectively) b.

Targeted gene expression on these MYB candidates was performed on the full developmental series of the three apple genotypes (Fig. 5c). RU displayed an upregulation of MYB52 (MD10G1010900) and MYB67 as compared to GD and SM from 40 DAFB, in a similar fashion to MdMYB93^23^ and MdOSC5. Little expression was observed at 20 and 31 DAFB for these MYBs, corroborating the absence of suberin (and thereby suberin-associated wax) in the skin cross-sections at these time points. MYB66 expression was also significantly increased in RU from 76 DAFB (as observed for lupane-type triterpene concentrations, Fig. 5a), although it was expressed from 20 DAFB in all genotypes.

### Candidate MYB promoters contain hormone-related *cis-*elements

To understand the role of these MYBs and how their transcription could be activated, we analysed the gene promoter sequences and predicted several cis-acting regulatory elements involved in the responsiveness to various hormones such as abscisic acid, methyl-jasmonate, gibberellin, salicylic acid and auxin, as well as MYB, MYC (bHLH) and WRKY binding sites (Table S4). All but MYB52-like contained ABRE cis-elements in their promoter region, indicating they may be involved in ABA response. MYB52, MYB52-like and MYB93 promoters displayed several MeJa-RE sites.

Interestingly, MYB52 contained an MBS MYB-binding site CAACTG, which is involved in drought stress-induced gene expression. The MYB52 promoter also contained 13 MYC-binding elements, suggesting an induction by bHLH TFs.

### Differential abscisic acid-related responses

Numerous ABA-responsive and ABA signalling pathway genes were affected by russeting (Fig. S5). The first committed and limiting step of ABA biosynthesis in plants is the oxidative cleavage of 9-cis-epoxycarotenoids by NCED (9-cis-epoxycarotenoid dioxygenase)^44^. One gene model encoding NCED3 (MD10G1194200) was upregulated in RU, whereas three NCED4 were downregulated. Interestingly, the NCED3 gene is highly induced by drought stress and regulated by an AP2/B3 TF (NGATHA1 (NGA1))^45^. Two ortholog genes of NGA1 (MD01G1091800, MD07G1162400) were found in our dataset and were also upregulated in RU, indicating that RU was possibly affected by drought stress. In contrast, genes coding for ABA receptors PYR/PYL/RCAR were generally downregulated, while genes from the rest of the core ABA signalling pathway, including PP2C phosphatases and SnRK2 kinases, were upregulated in RU. Genes coding for ABF2, one of the master TFs in abiotic stress-related ABA signalling^46^, displayed increased expression in RU. The expression of ABA-dependent drought-responsive genes such as the dehydrins RAB18 (MD02G1139900 and MD02G1140100), KIN2 (MD09G1079600) and RD29F (MD07G1268800)^47^, were also increased in RU compared with GD. ABA-dependent cutin and wax-related genes^12^ were all downregulated in RU. Finally, RD22 (another ABA-dependent drought-responsive gene) was also less expressed in RU along with its co-expressed TF, MYB15^48^.

### MYB52 and MYB66 activates the MdOSC5 promoter

**Fig. 6:**
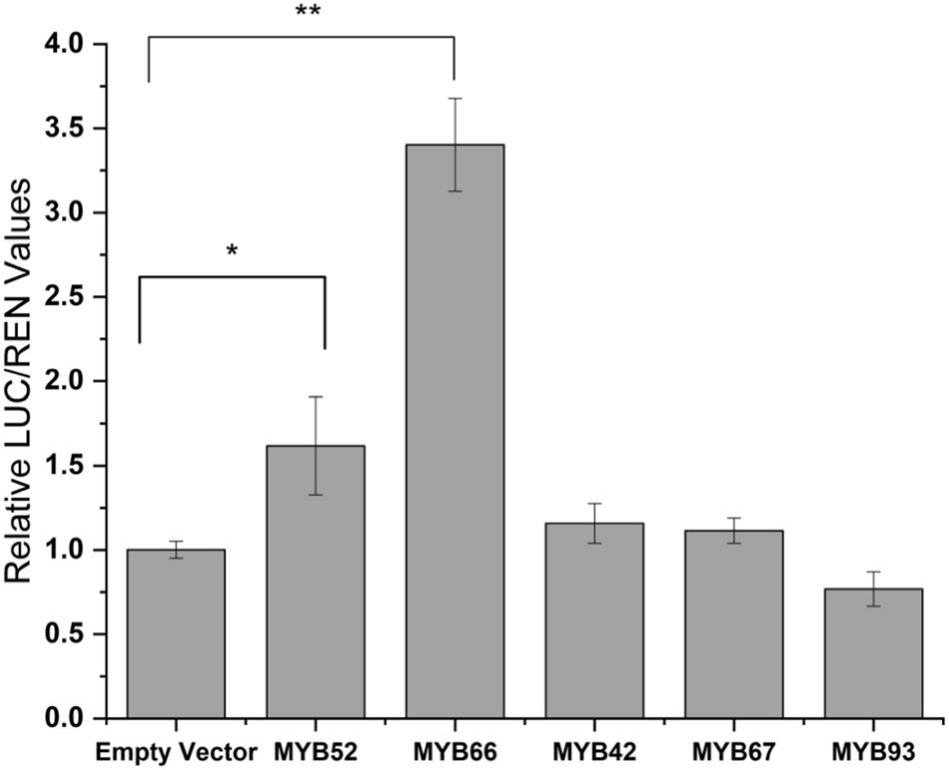
Activation of MdOSC5 promoter by MYBs. Promoter activity is expressed as a ratio of MdOSC5 promoter-luciferase (LUC) to 35S Renilla (REN), where an increase in activity equates to an increase in LUC relative to REN. Error bars are SE from three independent experiments (*n* = 12). Significance at the *p* < 0.05 (*) or *p* < 0.01 (**) levels were calculated according to student *t*-tests (*p* < 0.05)

Seven gene models encoding OSCs were directly related to the lupane-type triterpene concentrations (correlation coefficient above 0.9) (Fig. S6). Among them, MD17G1248500 (MdOSC5) was the most highly expressed, further suggesting its role in suberin-associated triterpene synthesis^25^. Based on the PlantCARE analysis, the MdOSC5 promoter contained MYBREs (Table S5). Using transactivation assays, we observed significant inductions of the promoter of MdOSC5, by MYB52 (1.62–fold) and to a larger extent by MYB66 (3.4-fold), when compared with the LUC activity in leaves co-transfected with empty vector control or the other MYBs included in the analysis (Fig. 6). These results indicate that MYB52 and MYB66 are potential activators of lupane-type triterpene biosynthesis.

## Discussion

### Reduced cuticle thickness associated with russeting

The cuticle plays a vital role in the protection of fruit, preserving the integrity and quality during development and postharvest storage. The sigmoidal increase of apple fruit size we observed during the early stages of growth (Fig. 1) corresponded to the peak rate of the cell expansion phase^49^ and triggers ongoing tensions in the cuticle^15^. Wax and cutin deposition must keep pace with increases in surface area to protect the underlying tissues from desiccation and pathogen attack^50^. If this primary skin fails, inducing microcracks, or is damaged due to environmental conditions, the barrier functions are restored by the formation of a ‘secondary’ fruit surface (periderm), created by the deposition of suberin in the inner part of the cell wall of epidermal cells and expressed as russeting on the apple skin. In our study, the apple genotypes GD and SM presented a smooth intact skin (Fig. 2a) and an increasing cuticle thickness throughout development (Fig. 2b). In contrast, RU showed a reduced cuticle thickness, variable epidermal cell sizes at 31 DAFB (Fig. 2a, b), and a compromised cuticle and incidence of suberin from 40 DAFB (Fig. 2a). These observations aligned with decreased expression of the primary cuticle biosynthesis TF, MdSHN3, and an increased expression of the suberin regulator MdMYB93 in RU (Fig. 4a, b).

The reduced cuticle thickness in RU appeared to be associated with microcracks and russeting (Fig. 2a). This was accompanied by the decrease of most genes involved in cuticle deposition, i.e. the wax/cutin pathways (Fig. S4) from 40 DAFB. The origin of the cuticle failure in russet apples is likely to be dependent on the cultivar, as suggested by contrasting QTL analyses^14,22^.

Cuticle formation is dynamic, with composition and coverage changing in response to environmental conditions, such as light/dark, ultraviolet, radiation, and drought^51,52^. Martin et al.^13^ suggested that ABA intrinsically regulates cuticle-formation during tomato leaf development, while abiotic stress such as drought exerts a secondary level of ABA-mediated control. Numerous ABA-dependent drought-responsive genes such as the dehydrins were also increased in RU compared with GD from 40 DAFB (Fig. S5), indicating a higher level of drought stress in RU, which could be partially due to the reduced cuticle layer. A recent transcriptome study in cucumber suggested that the decreased expression of cutin and wax biosynthetic genes might be responsible for sensitivity to drought^53^. In tomato and Arabidopsis leaves, ABA and drought stimulated cuticle formation, and thereby the expression of cutin and wax genes^54^. Nevertheless, we observed the opposite trend in RU skin (Fig. S5), where the responses to drought stress in an ABA-dependent manner were in favour of the suberin pathway, not cutin-wax biosynthesis. It is worth mentioning that, besides ABA, the phytohormones ethylene and gibberellin may also play a role in fruit cuticle development. The application of gibberellins during the early stage of fruit development has been shown to reduce cuticle microcracking and russeting in GD, via decreasing epidermal cell surface and fruit surface tensions^54,55^. This suggests a close relationship between hormone response, fruit growth, cuticle deposition, and russeting. Investigation of the hormonal pool might be informative in elucidating the onset of russeting.

The phenolic accumulation data suggests a remobilisation of phenolic building blocks from the phenylpropanoid and flavonoid pathway towards suberin and lignin biosynthesis as previously demonstrated in a transient experiment over-expressing a suberin regulator in tobacco leaves (Fig. S4)^23^. Similarly to our study, Bussato et al.^27^ showed that phloridzin content was higher in RU than in GD, both at 74 DAFB and harvest and there is now growing evidence that phloridzin accumulation is linked to suberin deposition in apple skin tissues^2,32^. This may shed further light on the regulation processes of the biosynthesis of this therapeutically interesting compound.

### The onset of russeting accompanied by a shift in triterpenes

The level of cutin-associated triterpenes (ursane and oleanane-type triterpenes) increased until 76 DAFB and remained constant throughout development, suggesting a role in maintaining cuticle strength, particularly in the early stages of development. In the impaired RU cuticle, the concentration of cutin-associated triterpenes remained low and stable throughout the developmental series, whereas those of lupane-types triterpenes increased from 76 DAFB, causing a shift in triterpene profile (Fig. 5a). It appears that betulinic acid and derivatives are preferentially produced in suberin-associated wax as compared to cutin-associated wax. Betulinic acid is slightly more lipophilic as compared to ursolic and oleanolic^5^, but how this triterpene change affects the structure of the cuticle and its permeability remains unanswered. The accumulation of triterpene-hydroxycinnamate conjugates is also intriguing. Since they accumulate in the same tissues, it is tempting to associate alkyl-hydroxycinnamates and triterpene-caffeate synthesis as some of the enzymatic reactions producing these compounds are similar. It is not known, however, whether they share a common synthesis pathway or regulatory networks.

The pathway leading to increases in betulinic acid and its caffeates was induced in RU from 40 DAFB, as attested by the expression of lupeol synthase MdOSC5. At 31 DAFB, the expression of MdSHN3 was not differentially regulated between the three genotypes studied, as opposed to MdOSC1/3 and MdOSC5, which were already downregulated and upregulated in RU, respectively, indicating downregulation of cutin-associated wax production and upregulation of suberin production. This suggests other regulatory pathways for both cutin-associated and suberin-associated wax biosynthesis in the early stage of development. Genes from the mevalonate pathway, such as the ones coding for 3-hydroxy-3-methylglutaryl-coenzyme A reductase (HMGR) (Fig. S4), were also downregulated in RU at the early time point. In *Medicago truncatulata*, HMGR1 is the rate-limiting enzyme for triterpene saponin biosynthesis^56^, indicating a potential key role for this enzyme in apple as well. Recent studies showed that AtMYB16, together with APETALA-domain TFs SHINE 1 (SHN1) coordinately regulate the cuticle synthesis^12,57^. AtMYB17 is closely related to AtMYB16, but its role in the cuticle deposition process is still elusive^58^, whereas MYB94 has been shown to positively regulate cuticular wax in *Arabidopsis*^59^.

### MYB regulation of the lupane-type triterpenes

Only a few TFs modulating plant terpene biosynthesis have been identified to date. Canonical correlation analysis on metabolite and RNA-seq data highlighted MYB52, MYB66, and MYB67 as potential lupane-type triterpene regulators. They could also be partially responsible for the induction of the suberin pathway. They all contained an ABRE motif in their promoter region, indicating possible ABA mediation. MYB67 has been associated with suberized tissues in apple, tomato, potato, Arabidopsis seed tissues and poplar^21,60,61^, but its functions remain unclear. Transient overexpression of the suberin regulator MdMYB93 induced also overexpression of MYB67 in tobacco^23^. MYB52 has previously been linked with secondary cell wall lignification in *Arabidopsis*^62^. AtMYB52 is also involved in ABA response and may confer drought tolerance^63^. Similar to MYB67, it was also upregulated in russet apples as compared to non-russet ones in a previous study (MDP0000291518^21^) but was not functionally characterised. MYB66 or WEREWOLF TF is a master regulator of root epidermal cell patterning in *Arabidopsis*, but its role in apple russeting (MD09G1183800 and MDP000124555^21^) is still elusive and requires further investigation. We found that MYB66, and MYB52 to a lesser extent, activated the promoter of OSC5, suggesting a regulatory role in the production of lupane-type triterpenes. They belong to the R2R3-MYB subgroups 15 (involved in the development and determination of cell fate and identity) and 21 (including MYBs connected with secondary cell wall biosynthesis)^64^ (Fig. S7). A recent tissue-specific transcriptomic study performed on birch stem identified an ortholog of MYB66 as potentially involved in betulin biosynthesis in phellem tissues^65^, supporting our new finding.

**Fig. 7:**
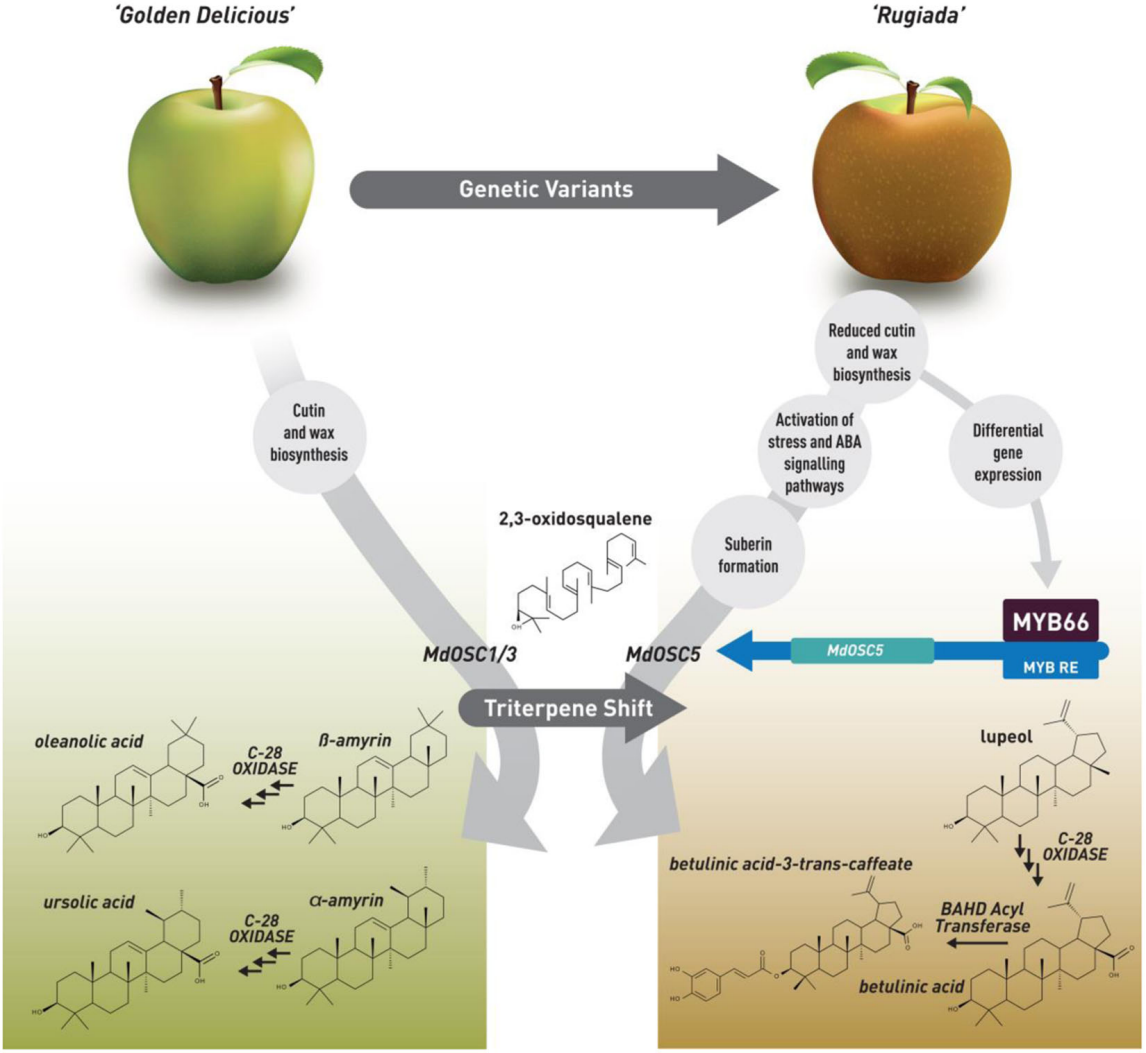
Model describing the events leading to a shift in triterpene metabolism during suberization of the skin of the apple cultivar ‘Rugiada’, a mutational sport of ‘Golden Delicious’. The transcription factor MdMYB66 binds to a response element (RE) in the promotor region of MdOSC5, which induces the production of lupane-type triterpenes in russet skin as opposed to the ursane-type and oleanane-type dominating in smooth waxy skin. OSC, oxydosqualene cyclase; BAHD Acyl-transferase, hydroxycinnamoyl-CoA transferase; C-28 oxidase = Cytochrome P450 CYP716A15

Taken together, the results of our study suggest the following scenario (summarised in Fig. 7): genetic variations in RU lead to a failure in early cuticle formation, generating microcracks. As the skin loses integrity, the fruit senses drought and hormonal signalling (likely ABA) triggers the formation of a periderm, including suberin, and a shift in triterpene profile to maintain the protective structure and resistance to pathogens. Our results suggest that a group of MYB transcription factors are important regulators of russet formation and that MYB66 is, at least in part, involved in the activation of the biologically active lupane-series of triterpenes. This study sheds further light on triterpene regulation and in view of the potential dietary health benefits of these molecules, altering triterpene content or composition of apple may become the focus of targeted breeding^4^.

## Materials and methods

### Plant material

Apples used in this study were GD (clone B) and its bud sports SM and RU. Trees were fifteen years old, grafted onto dwarfing rootstock M.9, and planted at the experimental farm of The University of Udine (Italy) (46°01'55.1“N 13°13'21.2“E), grown according to local common practices, avoiding any chemicals for russet control. Samples (three replicates of 6 to 9 fruit) were collected at flowering (06.04.2012, T0) and then at 10 fruit development stages corresponding to selected days after full bloom (DAFB, where the full bloom was defined as 100% open flowers) in 2012 (Table S6).

### Microscopy

Apple sections containing skin (4 × 4 × 4 mm) were fixed in 4% formaldehyde solution and embedded into wax as previously reported^66^. Histochemistry to determine the presence of lipids and lignin was carried out on 10 µm thick sections using Sudan IV (as described^67^) or phloroglucinol (2% [w/v] in 50% hydrochloric acid) for 30 min, at room temperature. Sections were observed with an Olympus Vanox AHBT3 (Olympus Optical Co Ltd., Tokyo, Japan) microscope using bright-field for stained tissues or ultraviolet light (excitation 330–385 nm, dichroic mirror 400 nm, emission ≥420 nm) for autofluorescence on unstained sections. Images were captured by an Olympus colour camera DP74 (Olympus Optical Co Ltd., Tokyo, Japan). Cuticle thickness analysis was carried out on images from cross-sections stained with Sudan IV using the ImageJ software (https://imagej.nih.gov/ij/). Ten measurements per fruit were taken and at least three fruit per time point.

### RNA extraction

Total RNA was extracted from 0.2 g of apple skin with the ‘Spectrum Plant total RNA’ (Sigma-Aldrich, St. Louis, MO, USA). The manufacturer’s protocol was modified by the addition of a spatula tip (5-mm width × 2 mm length) of PVPP to the ground tissue at the beginning of the extraction. The RNA was cleaned using the RNeasy MinElute Cleanup (Qiagen, Leusden, The Netherlands). RNA quantity, integrity and purity were assessed by NanoDrop 1000 Spectrophotometer (Thermo Scientific, Villebon-sur-Yvette, France), and 2100 Bioanalyzer system (Agilent Technologies, Santa Clara, CA, USA).

### RNA sequencing

GD and RU samples at developmental sampling time points T3 (40 DAFB), T6 (76 DAFB) and T8 (110 DAFB) were chosen for RNA-seq analysis. Three biological replicates for each cultivar at each time point were submitted for stranded mRNA library preparation using the TruSeq RNA Sample Prep Kit v 2.0 (Illumina). Libraries were quantified with a 2100 Bioanalyzer system (Agilent Technologies, Santa Clara, CA, USA). Sequencing was performed at IGA Technology Services (Udine, Italy), using 16-plex run on Illumina Hiseq2000 platform (Illumina, San Diego, CA, USA), 2 × 100 bp with >15 million reads per sample. Bioinformatic quality control pipeline and analysis methods are detailed in Methods S1. For building pathways, gene sequences provided by KEGG were blasted against the GDR (Genome Database for Rosaceae) blastn tool using the apple GDDH13 v1.1 chromosome database. Asterisks represent significant values at *p* < 0.01. KEGG pathway codes used were: 00940, 00941, 00900, 00909, 00062, and 00073.

### Real-time qPCR analysis

Quantitative Polymerase Chain Reaction (qPCR) was used to select key time points for RNA-seq data with a set of candidate genes for triterpene and russeting regulation. Primers were obtained from previous literature data or designed for new candidates using Geneious 10.0.9 (Table S7). mRNA retrotranscription was performed with the QuantiTect Reverse Transcription kit (Qiagen, Milan, Italy). qPCR was carried out on a CFX96 Touch Real-Time PCR Detection System (Bio-Rad, Milan, Italy) in a 10 µl reaction containing 2.5 µl of 40-fold diluted cDNA, 5 µl of SsoFast EvaGreen Supermix (BioRad), 1.5 µl of 2 µM forward and reverse primers, and 1 µl of RNAse/DNase free water. Conditions were: initial denaturation at 95 °C for 3 min, followed by 45 cycles of 94 °C for 15 s, annealing temperature of 60 °C for 20 s, 68 °C for 30 s, melting curve with 0.5 °C increments from 65 °C to 95 °C, and a final extension at 68 °C for 5 min. Relative gene expression of the target gene was calculated with the 2^−ΔΔCt^ method using *MdEF1A* as a reference gene.

### Triterpene and phenolic metabolite profiling

The extractions were performed on the basis of a previously described methodology^8^. Briefly, powdered freeze-dried skin material (40 mg) was rehydrated with 100 µl of water and mixed with ethyl acetate:hexane (1.5 ml, 50:50, *v/v*). The supernatant was evaporated to dryness and the pellet was re-extracted using ethanol:H_2_O (1 ml, 80:20, *v/v*). The supernatant was collected, combined with the lipophilic dried extract, and evaporated to dryness. Triterpenes and phenolic compounds were resuspended in MeOH: water (1 ml, 90:10, v/v) and filtered through a 0.2 µm PVDF filter. Each biological replicate of skin (three per time point and genotype) was extracted in duplicate (technical replicates). Each reported concentration was the average of six values. Details for triterpene and phenolic analysis protocols are described in Methods S2.

### Promoter analysis of MYB genes in apple

We extracted 2000 bp long sequences upstream of the transcription start site of the MYB genes from the apple genomic sequence (GDDH13_1-1_formatted.fasta), and then used PlantCARE (http://bioinformatics.psb.ugent.be/webtools/plantcare/html/) to predict cis-acting elements.

### Promoter cloning and analysis

PCR-based OSC promoter isolation was performed with ‘Royal Gala’ gDNA for OSC1 (Genbank number FJ032006, apple gene model number MD09G1167700) and OSC5 (KT383436, MD17G1158300) using primers listed (Table S8). Promoter fragments were inserted into the cloning site of pGreenII 0800-LUC as previously described^68^.

### Transactivation assays

All the constructs were transformed into *Agrobacterium tumefaciens* GV3101 and transient assays were carried out in *Nicotiana benthamiana*, as previously reported^68,69^. The full-length coding sequences for apple MYB42, 52, 66, 67 and 93 were cloned as previously described^23^.

### Statistical analysis

Principal Component Analysis (PCA), Partial Least Squares Discriminant Analysis (PLS-DA), Canonical Correlation Analysis (CCA) and Analysis of Variance (ANOVA) followed by post-hoc difference analysis (Tukey’s LSD test, at 5% significance level) were conducted using R language version 3.5.1(R Core Team, 2015) and the “mixOmics” package version 6.6.0^70^.

## Supplementary information

Revised Supplementary Information

Fig S4

Fig S5

Supplementary Table S1

Supplementary Table S2

Supplementary Table S3

## Data Availability

RNA sequence raw data were deposited in the NCBI Sequence Read Archive within the BioProjects PRJNA663349 and PRJNA663351.
