# Supplementary material for: Differential regulation of triterpene biosynthesis induced by an early failure in cuticle formation in apple
Source: Hortic Res. 2021 Apr 1;8:75. doi: 10.1038/s41438-021-00511-4 (PMC8012369; doi:10.1038/s41438-021-00511-4)
Supplement: Supplementary file 1 — Revised Supplementary Information [file 41438_2021_511_MOESM1_ESM.docx]

**Supplementary Information**

*Differential regulation of triterpene biosynthesis induced by an early failure in cuticle formation in apple.*

This document includes Figure S1, S2, S6, S7, S8, Tables S4 to S8 and Method S1 and S2.

**Figure S1:** Cross sections of the epidermal layer of ‘Golden Delicious’ and its russet and non-russet clones, ‘Rugiada’ and ‘Smoothee’, respectively, during fruit development.

**Figure S2:** Total phenolic contents in the three different apple varieties under investigation.

**Figure S3**: Statistical analyses of the transcriptomics and metabolite data.

**Figure S4**: Schematic heatmap reconstituting cutin, wax, suberin, phenylpropanoid and (tri)terpenoid pathways and their differential gene expression between ‘Rugiada’ and ‘Golden Delicious’. (Separate file).

**Figure S5:** Reconstituted abscisic acid (ABA) pathway and target genes including RNA-Seq expression data. (Separate file).

**Figure S6:** Heatmap representing the regularised canonical correlation (rCC) coefficients between triterpene concentrations and expression of OSCs genes.

**Figure S7:** Phylogenetic analysis of the apple and Arabidopsis MYB transcription factors.

**Table S1**: Phenolic compound and triterpene concentrations in the three contrasting apple varieties ‘Rugiada’, ‘Golden Delicious’ and ‘Smoothee’. (Separate spreadsheet).

**Table S2:** Genome-wide analysis of specific gene families. (Separate spreadsheet).

**Table S3**: List of the 91 MYBs differentially expressed in the RNA-seq dataset comparing the russet apple variety ‘Rugiada’ and the non-russet ‘Golden Delicious’. (Separate spreadsheet).

**Table S4**: Putative cis-acting regulatory elements identified in the promoter sequence of MdOSC1, MdOSC5, MYB66, MYB67, MYB52 and MYB93.

**Table S5**: Putative cis-acting regulatory elements identified in the promoter sequence of MYB66, MYB67, MYB52, MYB93, MdOSC1, and MdOSC5.

**Table S6:** Apple sample collection time points.

**Table S7**: List of the primers used for the Reverse Transcriptase-quantitative Polymerase Chain Reaction (RT-qPCR) analysis.

**Table S8**: List of the primers used for the isolation of MdOSC1 and MdOSC5 promoter region.

**Method S1:** RNA sequencing and data analysing**.**

**Method S2:** Metabolite profiling.

**Supplementary Figures**

**Figure S1:**  **Cross sections of the epidermal layer of ‘Golden Delicious’ and its russet and non-russet clones, ‘Rugiada’ and ‘Smoothee’, respectively, during fruit development**. (a) Light microscopy of the epidermal layers under the lipid stain Sudan IV, indicating the thickness of the cuticle (pink) as well as the presence of suberin in the periderm of ‘Rugiada’. (b) Phloroglucinol staining of the fruit sections showing lignified cell-wall tissue in the periderm (pink and red) from T6 in ‘Rugiada’ and T8 in russeted patches of ‘Golden Delicious’. Scale bar = 50 µm. DAFB: Days After Full Bloom


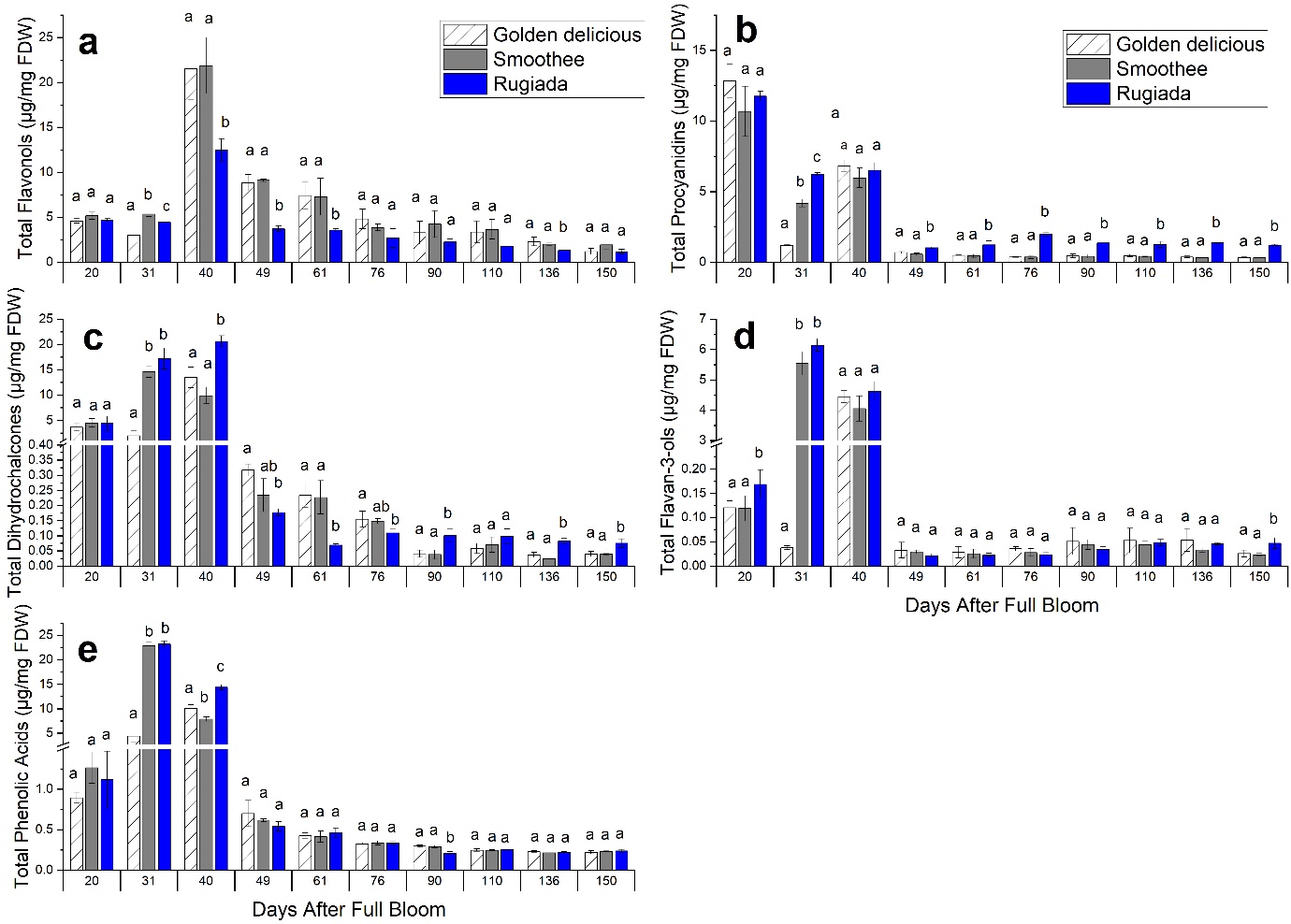


**Figure S2:** Total phenolic contents in the 3 different *Malus* x *domestica* varieties under investigation (See Table 1 for correspondence of T1 to T10 in Days after Full Bloom (DAFB)): ‘Golden Delicious’, and its fully russeted and russet-resistant mutational sports, ‘Rugiada’ and ‘Smoothee’, respectively. Significance was calculated according to one-way ANOVA of p < 0.05 per time point, where lowercase letters above bars result from the comparison of groups using Tukey’s test (for phenolics A to E). Groups connected by the same letter are not significantly different. Data are expressed per µg per mg of freeze-dried weight (FDW). Values at 20 and 31 DAFB are expressed per mg of total fruit and not skin, as the fruit were too small at this stage to peel them consistently.


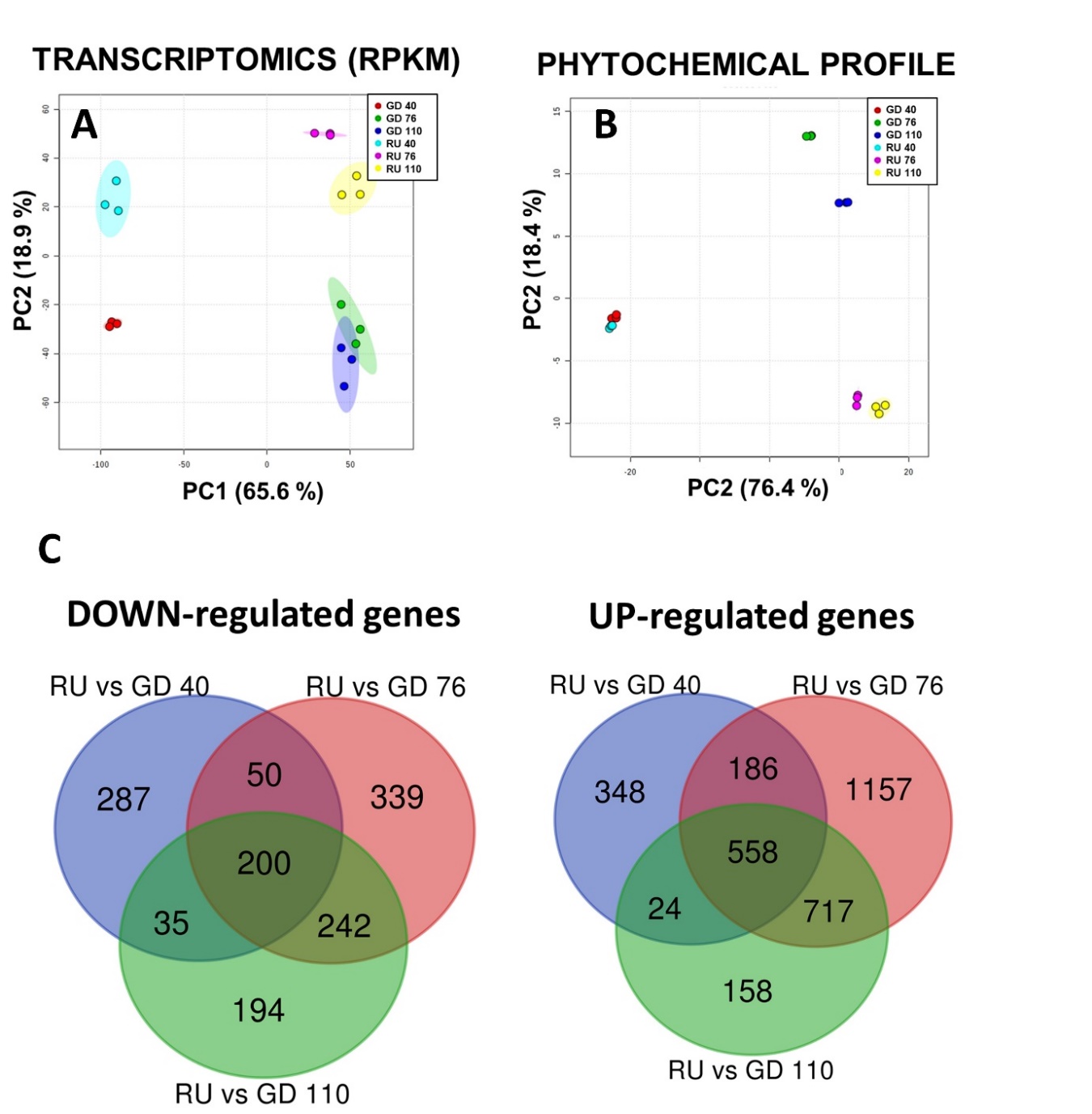


**Figure S3**: Statistical analyses of the transcriptomics data comparing the skin of ‘Golden Delicious’ (GD) and the one of ‘Rugiada’ (RU) at three different time points (T): 40 Days after Full Bloom (DAFB) (T3), 76 DAFB (T6) and 110 DAFB (T8). (A) Principal Component Analysis (PCA) of the RPKM of all genes identified. (B) PCA of the metabolite/phytochemical data. (C) Venn diagram of differentially expressed genes (DEG; up- and down-regulated genes) in the different comparative groups. Venn diagram was drawn using a tool developed by VIB/UGent (<http://bioinformatics.psb.ugent.be/webtools/Venn/>).


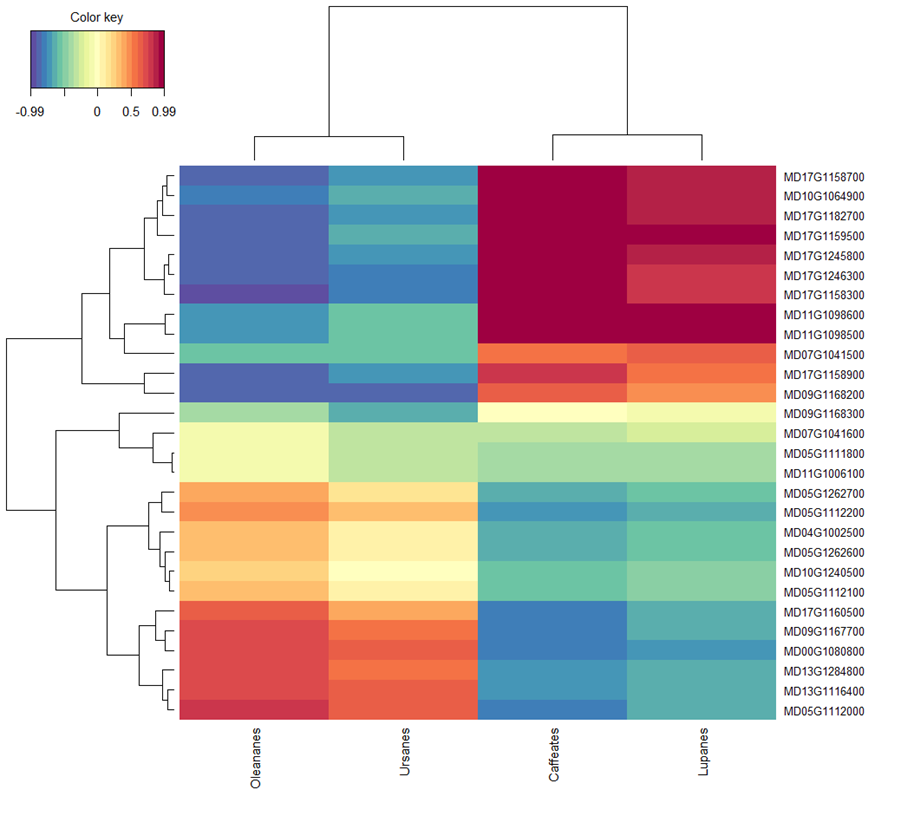


**Figure S6:** Heat map representing the regularised canonical correlation (rCC) coefficients between triterpene concentrations (classified per type) and the expression of genes identified as OXIDOSQUALENE CYCLASES (OSCs) (RPKM values). The heat map was calculated using normalized log10-transformed metabolite/gene expression levels, mean centred, and standardised.


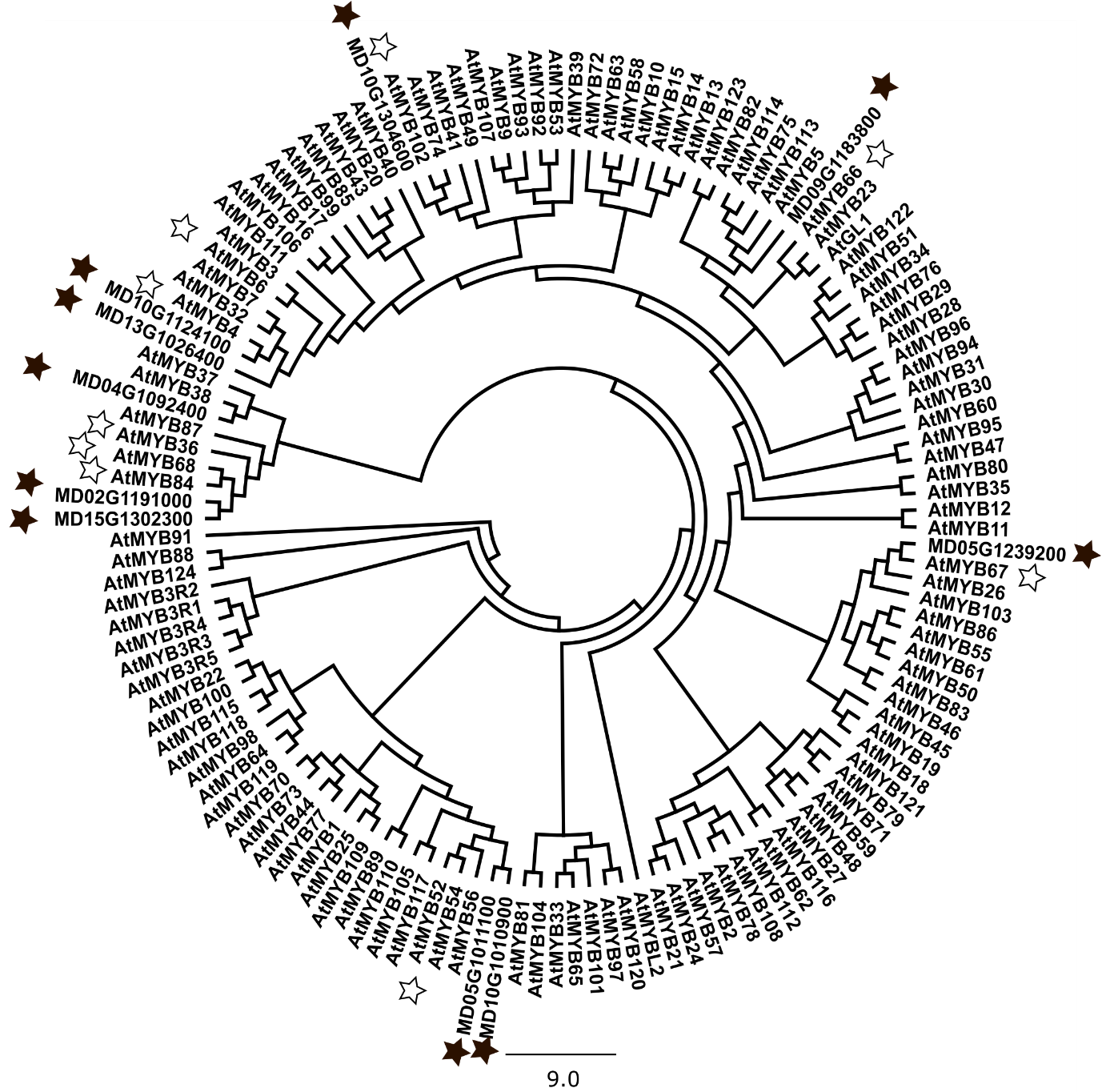


**Figure S7:** Phylogenetic analysis of the apple MYB transcription factors which were differentially regulated between russeted and non-russeted apple skins (black stars) and identified in cluster II (Figure 5b). The amino acid sequences of these genes were compared to MYB transcription sequences identified in Arabidopsis using the Geneious tool. White stars indicate the best BLAST match of the apple gene models. The cladogram was generated using UPGMA algorithm and Jukes-Cantor genetic distance model.

**Table S4**: Putative cis-acting regulatory elements identified in the promoter sequence of MdOSC1, MdOSC5, MYB66, MYB67, MYB52 and MYB93. Numbers represent *cis*-acting element numbers on the plus chain/minus chain. *Abbreviations*: ABRE: cis-acting regulatory element (RE) involved in the abscisic acid (AB)-responsiveness; MeJa-RE: cis-acting regulatory element (RE) involved in the methyl-jasmonate (MeJa)-responsiveness; RE: cis-acting regulatory element (RE).

**Table S5**: Putative cis-acting regulatory elements identified in the promoter sequence of MYB66, MYB67, MYB52, MYB93, MdOSC1, and MdOSC5. The numbers in the table represent *cis*-acting element numbers on the plus chain/minus chain. *Abbreviations*: MBS: MYB-binding site involved in drought-inducibility. MYC: bHLH transcription binding site. W-Box: WRKY transcription factor binding site.

| **Gene ID** | **NAC binding site** | |  | **MYB binding sites** | | | | | |  | **MYC** | | |  | **W-Box** |
| --- | --- | --- | --- | --- | --- | --- | --- | --- | --- | --- | --- | --- | --- | --- | --- |
|  | ACACGT | ACGTGT |  | CAACCA | CAACAG | CCGTTG | TAACTG | CAACTG (MBS) | TAACCA |  | TCTCTTA | CATTTG | CATGTG |  | TTGACC |
| MYB66 (MD09G1183800) | 0/2 | 0/1 |  | 2/0 | 0/1 | 1/1 | 1/0 | 0/0 | 0/0 |  | 1/0 | 1/0 | 1/1 |  | **0/1** |
| MYB67 (MD05G1239200) | 0/1 | 0/0 |  | **0/1** | 0/0 | **1/0** | **0/1** | 0/0 | 0/0 |  | 0/0 | 2/3 | 0/0 |  | **0/1** |
| MYB52-like (MD05G1011100) | 0/0 | 0/0 |  | **1/1** | 0/0 | 0/0 | 0/0 | 0/0 | **0/1** |  | 0/0 | 2/1 | 0/0 |  | 0/0 |
| MYB52 (MD10G1010900) | 0/0 | 0/0 |  | 0/0 | **1/0** | 0/0 | 0/0 | **0/1** | 0/0 |  | 0/0 | **11/1** | 0/1 |  | 0/0 |
| MYB93 (MD15G1323500) | 0/0 | 0/0 |  | **1/3** | 0/0 | 0/0 | **1/0** | 0/0 | **0/3** |  | 1/0 | 0/1 | 0/2 |  | **1/1** |
| MdOSC1 | 1/0 | 0/1 |  | 1/0 | 0/0 | 0/0 | **2/2** | 0/0 | 0/0 |  | 1/0 | 0/0 | 0/0 |  | 0/1 |
| MdOSC5 | 0/0 | 0/1 |  | 1/0 | 0/1 | 0/0 | 2/0 | **1/2** | 0/0 |  | 0/1 | 1/0 | 0/1 |  | 0/0 |

**TableS6**: Apple sample collection at time points one to ten (T1-10) corresponding to dates and days after full bloom (DAFB)

**Table S7**: List of the primers used for the RT-qPCR analysis.

|  |
| --- |
|  |
|  |
|  |
| **Table S8**: List of the primers used for the isolation of MdOSC1 and MdOSC5 promoter region.   |

*3 bases changed to create NCO1 site for pGreen cloning.

**Method S1** **RNA sequencing and Data analysing**

Quality of RNA-Seq data was checked with FastQC/0.11.7 (Wingett & Andrews, 2018). Based on the QC report, adaptors were removed from raw sequencing data with fastq-mcf in the ea-utils packages, then further trimmed using fastx_trimmer in the FASTX-Toolkit (<http://hannonlab.cshl.edu/fastx_toolkit/index.html>) with a Phred score of 30 and the minimum length of 90 bp. The cleaned reads were mapped to the reference genome of *Malus* x *domestica* GDDH13_v1.1 (Daccord *et al.,* 2017) using bowtie2/2.2.9 with parameters ‘-q --very-sensitive-local -I 0 -X 500 --fr’ (Langmead & Salzberg, 2012). Split alignments were converted to BAM files using samtools/1.9. Mapping quality was accessed with QualiMap (Okonechnikov *et al.,* 2015). Read counts for genes in each replicate were extracted from the alignment files with sam2counts_galaxy.py in the NGS-RNA package (<http://www.htslib.org/>). Gene differential expression test were carried out with DESeq2 and ReportingTools in R/3.6.1 (Huntley *et al.,* 2013). Gene annotation information was retrieved from GDR/GDDH13 v1.1 Whole Genome Assembly & Annotation (<https://www.rosaceae.org>). Expression values were calculated using the Reads per Kilobase transcript per Million reads (RPKM) method (<https://www.rna-seqblog.com/rpkm-fpkm-and-tpm-clearly-explained/>).

**Method S2** **Metabolite profiling**

The extractions were performed as previously described ([Andre *et al.,* 2013](#_3znysh7)). Powdered freeze-dried skin material (40 mg) was first rehydrated with 100 µL of water and then mixed with ethyl acetate:hexane (1.5 mL, 50:50, *v/v*). This mixture was then homogenized using a vortex for 30 s and shaken for 1 h at room temperature. After centrifugation at *10,000 g* for 15 min, the supernatant was collected and evaporated to dryness using a centrifugal vacuum evaporator. The pellet was re-extracted using EtOH:H2O (1 mL, 80:20,v*/v*), homogenised, sonicated for 10 min on ice, shaken for 2 h at room temperature and centrifuged as above. The supernatant was collected, combined with the lipophilic dried extract, and evaporated to dryness. Triterpenes and phenolic compounds were resuspended in MeOH: water (1 mL, 90:10, v/v) and filtered through a 0.2 µm PVDF filter into a UPLC vial for triterpene analysis. Five hundred µL were further evaporated to dryness and resuspended in acetonitrile : water (500 µL, 1:99, *v/v*) for phenolic analyses. Each biological replicates of skin (3 per time points and genotypes) were extracted in duplicate (technical replicates). Therefore, each reported concentration is the average of six values.

*Triterpenoid Compound Analysis*

Extracts were analyzed with a Waters Acquity UPLC system (Milford, MA, USA) hyphenated to a Diode Array Detector (UPLC-DAD). The separation of the 5 µL aliquot was performed on a reverse-phase Acquity UPLC BEH C18 column (2.1 × 100 mm, 1.7 μm particle size, Waters, Milford, MA, USA). The eluents were 0.05 % o-phosphoric acid in water (A) and 0.05 % o-phosphoric acid in methanol (B). The gradient was as follows: 0 min, 75% B; 2 min, 75% B; 16 min, 82% B; 25 min, 100% B; 26.5 min, 100% B; 27 min, 75% B; 30 min, 75% B. The flow rate was of 0.3 mL min^-1^ and the column temperature was 40°C. Ursolic acid (UA), oleanolic acid (OA), betulinic acid (BA), corosolic acid, maslinic acid, lupeol, betulinaldehyde, betulin, α-amyrin, β-amyrin as well as BA-transCaffeate (BA-transC) were identified by their retention time and spectral data compared with authentic standards and were quantified at 210 nm (UA, OA, and BA) and 320 nm (BA-transC) using five-point calibration curves. Excellent linearity (R^2^ > 0.99) was obtained in the concentration range 100–6.25 µg mL^-1^ for all compounds. BA-cisCaffeate and OA-transCaffeate were quantified as BA-transC equivalent.

*Phenolic Compound Analysis*

The quantification of phenolic compounds was carried out using a Waters Acquity UPLC system (Milford, MA) equipped with a photodiode array detector (PDA). An aliquot of 5 µL was injected onto an Acquity UPLC HSS T3 column (2.1 x 100 mm, 1.8 µm particle size, Waters, Milford, MA) at 50°C and a flow rate of 0.7 mL/min. The eluents were: 0.1% formic acid in water (A) and 0.1% formic acid in acetonitrile (B). The gradient was as follows: 0 min, 1% B; 20 min, 8% B; 30 min, 14% B; 35 min, 20% B; 36 min, 100 % B; 40 min, 100 % B; 41 min, 1% B; 45 min, 1 % B. Phenolic compounds were detected at 280, 320, 360 or 520 nm according to their absorption maximum. For quantification, a five-point calibration curve was used. Furthermore, a validation standard was injected after every 10th injection. Flavanol derivatives were quantified as catechin equivalents, flavonols as quercetin-3-galactoside equivalents, hydroxycinnamic acid as chlorogenic acid equivalents, and dihydrochalcone derivatives as phloridzin equivalents.

**References**

**Andre CM, Larsen L, Burgess EJ, Jensen DJ, Cooney JM, Evers D, Zhang JL, Perry NB, Laing WA. 2013**. Unusual Immuno-Modulatory Triterpene-Caffeates in the Skins of Russeted Varieties of Apples and Pears. *Journal of Agricultural and Food Chemistry* **61:** 2773-2779.

**Daccord N, Celton JM, Linsmith G, Becker C, Choisne N, Schijlen E, van de Geest H, Bianco L, Micheletti D, Velasco R et al. 2017.** High-quality de novo assembly of the apple genome and methylome dynamics of early fruit development. *Nature genetics* **49:** 1099–1106.

**Huntley MA, Larson JL, Chaivorapol C, Becker G, Lawrence M, Hackney JA, Kaminker JS. 2013.** ReportingTools: an automated result processing and presentation toolkit for high-throughput genomic analyses. *Bioinformatics* **29**: 3220-3221.

**Wingett SW, Andrews S. 2018.** FastQ Screen: A tool for multi-genome mapping and quality control. F1000Research, 7.

**Okonechnikov K, Conesa A, García-Alcalde F. 2016.** Qualimap 2: advanced multi-sample quality control for high-throughput sequencing data. *Bioinformatics*, **32:** 292-294.
