# Supplementary material for: Differential regulation of triterpene biosynthesis induced by an early failure in cuticle formation in apple
Source: Hortic Res. 2021 Apr 1;8:75. doi: 10.1038/s41438-021-00511-4 (PMC8012369; doi:10.1038/s41438-021-00511-4)
Supplement: Supplementary file 2 — Fig S4 [file 41438_2021_511_MOESM2_ESM.pdf]

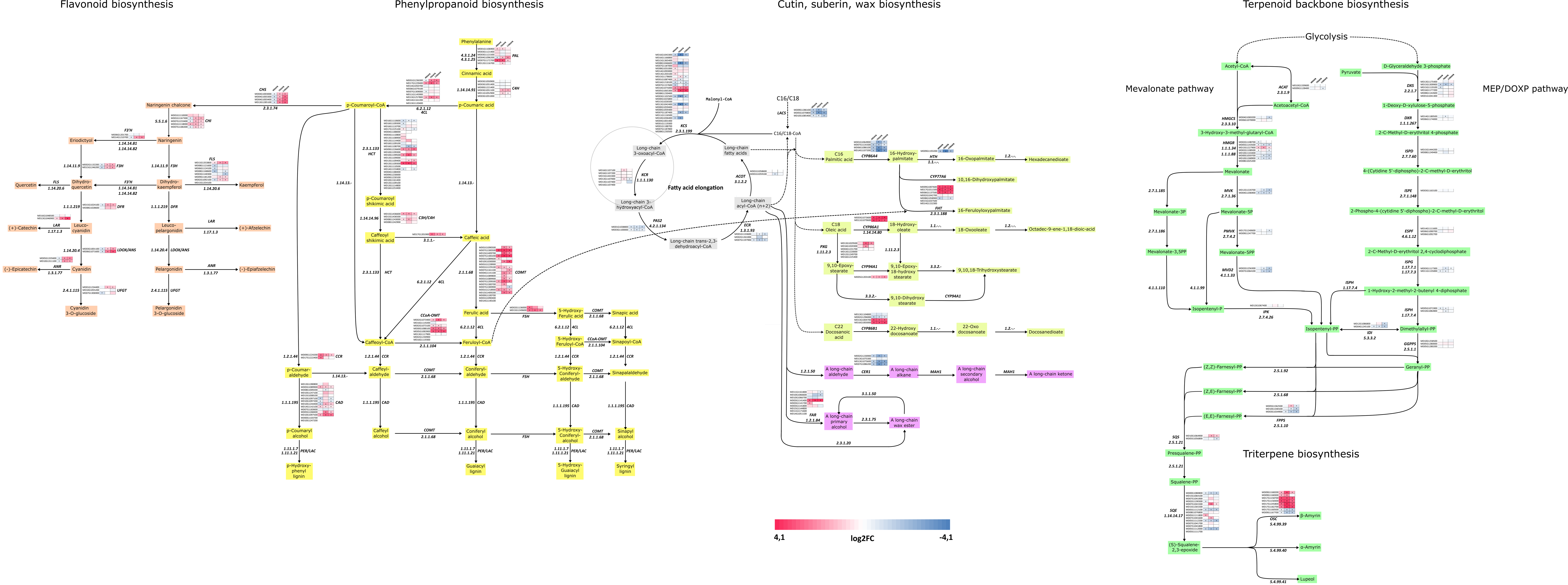

Flavonoid biosynthesis
Phenylpropanoid biosynthesis
Cutin, suberin, wax biosynthesis
Terpenoid backbone biosynthesis
Naringenin chalcone
CHS
2.3.1.74
5.5.1.6
CHI
F3'H
Eriodictyol
1.14.14.81
1.14.14.82
Naringenin
F3H
1.14.11.9
FLS
Quercetin
FLS
1.14.20.6
Dihydro-quercetin
Dihydro-kaempferol
Kaempferol
1.1.1.219
DFR
(+)-Catechin
LAR
1.17.1.3
Leuco-cyanidin
Leuco-pelargonidin
(+)-Afzelechin
1.14.20.4
LDOX/ANS
(-)-Epicatechin
ANR
1.3.1.77
Cyanidin
Pelargonidin
(-)-Epiafzelechin
2.4.1.115
UFGT
Cyanidin 3-O-glucoside
Pelargonidin 3-O-glucoside
Phenylalanine
4.3.1.24
4.3.1.25
PAL
Cinnamic acid
1.14.14.91
C4H
p-Coumaroyl-CoA
6.2.1.12
4CL
p-Coumaric acid
2.3.1.133
HCT
1.14.13.-
p-Coumaroyl shikimic acid
1.14.14.96
C3H/C4H
Caffeoyl shikimic acid
3.1.1.-
Caffeic acid
2.1.1.68
COMT
6.2.1.12
4CL
Ferulic acid
F5H
5-Hydroxy-Ferulic acid
Sinapic acid
CCoA-OMT
Caffeoyl-CoA
2.1.1.104
Feruloyl-CoA
5-Hydroxy-Feruloyl-CoA
Sinapoyl-CoA
1.2.1.44
CCR
p-Coumar-aldehyde
Caffeyl-aldehyde
Coniferyl-aldehyde
5-Hydroxy-Coniferyl-aldehyde
Sinapalaldehyde
1.1.1.195
CAD
p-Coumaryl alcohol
Caffeyl alcohol
Coniferyl alcohol
5-Hydroxy-Coniferyl-alcohol
Sinapyl alcohol
1.11.1.7
1.11.1.21
PER/LAC
p-Hydroxy-phenyl lignin
Guaiacyl lignin
5-Hydroxy-Guaiacyl lignin
Syringyl lignin
Malonyl-CoA
KCS
2.3.1.199
Long-chain 3-oxoacyl-CoA
KCR
1.1.1.130
Long-chain 3-hydroxyacyl-CoA
Fatty acid elongation
PAS2
4.2.1.134
Long-chain trans-2,3-dehydroacyl-CoA
ECR
1.3.1.93
Long-chain acyl-CoA (n+2)
ACOT
3.1.2.2
Long-chain fatty acids
C16/C18
LACS
C16/C18-CoA
C16 Palmitic acid
CYP86A4
16-Hydroxy-palmitate
HTH
1.1.-.-
16-Oxopalmitate
1.2.-.-
Hexadecanedioate
CYP77A6
10,16-Dihydroxypalmitate
FHT
2.3.1.188
16-Feruloyloxypalmitate
C18 Oleic acid
CYP86A1
1.14.14.80
18-Hydroxy-oleate
18-Oxooleate
Octadec-9-ene-1,18-dioic-acid
PXG
1.11.2.3
9,10-Epoxy-stearate
CYP94A1
9,10-Epoxy-18-hydroxy stearate
3.3.2.-
9,10,18-Trihydroxystearate
9,10-Dihydroxy stearate
C22 Docosanoic acid
CYP86B1
22-Hydroxy docosanoate
22-Oxo docosanoate
Docosanedioate
1.2.1.50
A long-chain aldehyde
CER1
A long-chain alkane
MAH1
A long-chain secondary alcohol
A long-chain ketone
3.1.1.50
FAR
1.2.1.84
A long-chain primary alcohol
2.3.1.75
A long-chain wax ester
2.3.1.20
4,1
log2FC
-4,1
Glycolysis
Acetyl-CoA
Pyruvate
D-Glyceraldehyde 3-phosphate
Mevalonate pathway
MEP/DOXP pathway
ACAT
2.3.1.9
Acetoacetyl-CoA
DXS
2.2.1.7
1-Deoxy-D-xylulose-5-phosphate
HMGCS
2.3.3.10
DXR
1.1.1.267
3-Hydroxy-3-methyl-glutaryl-CoA
2-C-Methyl-D-erythritol 4-phosphate
HMGR
1.1.1.34
1.1.1.88
ISPD
2.7.7.60
Mevalonate
4-(Cytidine 5'-diphospho)-2-C-methyl-D-erythritol
2.7.1.185
MVK
2.7.1.36
ISPE
2.7.1.148
Mevalonate-3P
Mevalonate-5P
2-Phospho-4-(cytidine 5'-diphospho)-2-C-methyl-D-erythritol
2.7.1.186
PMVK
2.7.4.2
ESPF
4.6.1.12
Mevalonate-3,5PP
Mevalonate-5PP
2-C-Methyl-D-erythritol 2,4-cyclodiphosphate
MVD2
4.1.1.33
ISPG
1.17.7.1
1.17.7.3
4.1.1.110
4.1.1.99
ISPH
1.17.7.4
1-Hydroxy-2-methyl-2-butenyl 4-diphosphate
Isopentenyl-P
IPK
2.7.4.26
Isopentenyl-PP
IDI
5.3.3.2
Dimethylallyl-PP
GGPPS
2.5.1.1
(Z,Z)-Farnesyl-PP
2.5.1.92
Geranyl-PP
(Z,E)-Farnesyl-PP
2.5.1.68
(E,E)-Farnesyl-PP
FPPS
2.5.1.10
SQS
2.5.1.21
Presqualene-PP
Triterpene biosynthesis
2.5.1.21
Squalene-PP
SQE
1.14.14.17
β-Amyrin
OSC
5.4.99.39
(S)-Squalene-2,3-epoxide
5.4.99.40
α-Amyrin
5.4.99.41
Lupeol
