# Supplementary material for: Differential regulation of triterpene biosynthesis induced by an early failure in cuticle formation in apple
Source: Hortic Res. 2021 Apr 1;8:75. doi: 10.1038/s41438-021-00511-4 (PMC8012369; doi:10.1038/s41438-021-00511-4)
Supplement: Supplementary file 3 — Fig S5 [file 41438_2021_511_MOESM3_ESM.pdf]

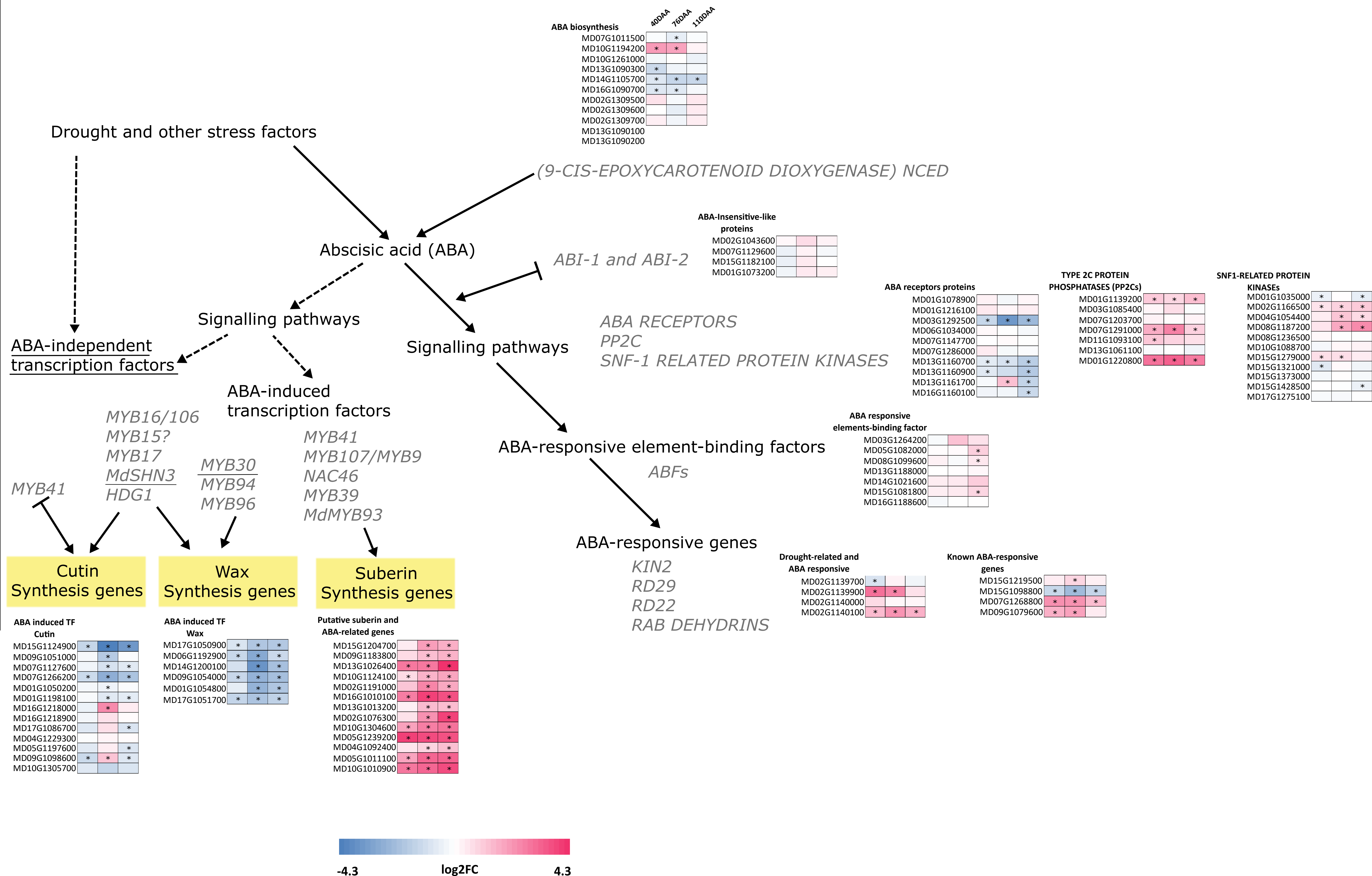

ABA biosynthesis
40DAA
76DAA
110DAA
MD07G1011500
MD10G1194200
MD10G1261000
MD13G1090300
MD14G1105700
MD16G1090700
MD02G1309500
MD02G1309600
MD02G1309700
MD13G1090100
MD13G1090200
Drought and other stress factors
(9-CIS-EPOXYCAROTENOID DIOXYGENASE) NCED
Abscisic acid (ABA)
ABI-1 and ABI-2
ABA-Insensitive-like proteins
MD02G1043600
MD07G1129600
MD15G1182100
MD01G1073200
Signalling pathways
Signalling pathways
ABA-independent transcription factors
ABA-induced transcription factors
ABA RECEPTORS
PP2C
SNF-1 RELATED PROTEIN KINASES
ABA receptors proteins
MD01G1078900
MD01G1216100
MD03G1292500
MD06G1034000
MD07G1147700
MD07G1286000
MD13G1160700
MD13G1160900
MD13G1161700
MD16G1160100
TYPE 2C PROTEIN PHOSPHATASES (PP2Cs)
MD01G1139200
MD03G1085400
MD07G1203700
MD07G1291000
MD11G1093100
MD13G1061100
MD01G1220800
SNF1-RELATED PROTEIN KINASEs
MD01G1035000
MD02G1166500
MD04G1054400
MD08G1187200
MD08G1236500
MD10G1088700
MD15G1279000
MD15G1321000
MD15G1373000
MD15G1428500
MD17G1275100
MYB16/106
MYB15?
MYB17
MdSHN3
HDG1
MYB41
MYB30
MYB94
MYB96
MYB41
MYB107/MYB9
NAC46
MYB39
MdMYB93
ABA-responsive element-binding factors
ABFs
ABA responsive elements-binding factor
MD03G1264200
MD05G1082000
MD08G1099600
MD13G1188000
MD14G1021600
MD15G1081800
MD16G1188600
ABA-responsive genes
KIN2
RD29
RD22
RAB DEHYDRINS
Drought-related and ABA responsive
MD02G1139700
MD02G1139900
MD02G1140000
MD02G1140100
Known ABA-responsive genes
MD15G1219500
MD15G1098800
MD07G1268800
MD09G1079600
Cutin Synthesis genes
Wax Synthesis genes
Suberin Synthesis genes
ABA induced TF Cutin
MD15G1124900
MD09G1051000
MD07G1127600
MD07G1266200
MD01G1050200
MD01G1198100
MD16G1218000
MD16G1218900
MD17G1086700
MD04G1229300
MD05G1197600
MD09G1098600
MD10G1305700
ABA induced TF Wax
MD17G1050900
MD06G1192900
MD14G1200100
MD09G1054000
MD01G1054800
MD17G1051700
Putative suberin and ABA-related genes
MD15G1204700
MD09G1183800
MD13G1026400
MD10G1124100
MD02G1191000
MD16G1010100
MD13G1013200
MD02G1076300
MD10G1304600
MD05G1239200
MD04G1092400
MD05G1011100
MD10G1010900
-4.3
log2FC
4.3
